# Comparison of *Fusarium graminearum* Transcriptomes on Living or Dead Wheat Differentiates Substrate-Responsive and Defense-Responsive Genes

**DOI:** 10.3389/fmicb.2016.01113

**Published:** 2016-07-26

**Authors:** Stefan Boedi, Harald Berger, Christian Sieber, Martin Münsterkötter, Imer Maloku, Benedikt Warth, Michael Sulyok, Marc Lemmens, Rainer Schuhmacher, Ulrich Güldener, Joseph Strauss

**Affiliations:** ^1^Fungal Genetics and Genomics Unit, Division of Microbial Genetics and Pathogen Interactions, Department of Applied Genetics and Cell Biology, BOKU University, University and Research Centre TullnTulln, Austria; ^2^Bioresources, Austrian Institute of Technology GmbHTulln, Austria; ^3^Department of Earth and Planetary Sciences, University of California, BerkeleyBerkeley, CA, USA; ^4^Helmholtz Zentrum München, Deutsches Forschungszentrum für Gesundheit und UmweltNeuherberg, Germany; ^5^Department for Agrobiotechnology (IFA-Tulln), BOKU UniversityTulln, Austria; ^6^Department of Genome-oriented Bioinformatics, Wissenschaftszentrum Weihenstephan, Technische Universität MünchenMünchen, Germany

**Keywords:** *Fusarium*, secondary metabolism, pathogenicity factors, defense genes, active plant, passive plant

## Abstract

*Fusarium graminearum* is an opportunistic pathogen of cereals where it causes severe yield losses and concomitant mycotoxin contamination of the grains. The pathogen has mixed biotrophic and necrotrophic (saprophytic) growth phases during infection and the regulatory networks associated with these phases have so far always been analyzed together. In this study we compared the transcriptomes of fungal cells infecting a living, actively defending plant representing the mixed live style (pathogenic growth on living flowering wheat heads) to the response of the fungus infecting identical, but dead plant tissues (cold-killed flowering wheat heads) representing strictly saprophytic conditions. We found that the living plant actively suppressed fungal growth and promoted much higher toxin production in comparison to the identical plant tissue without metabolism suggesting that molecules signaling secondary metabolite induction are not pre-existing or not stable in the plant in sufficient amounts before infection. Differential gene expression analysis was used to define gene sets responding to the active or the passive plant as main impact factor and driver for gene expression. We correlated our results to the published *F. graminearum* transcriptomes, proteomes, and secretomes and found that only a limited number of *in planta*- expressed genes require the living plant for induction but the majority uses simply the plant tissue as signal. Many secondary metabolite (SM) gene clusters show a heterogeneous expression pattern within the cluster indicating that different genetic or epigenetic signals govern the expression of individual genes within a physically linked cluster. Our bioinformatic approach also identified fungal genes which were actively repressed by signals derived from the active plant and may thus represent direct targets of the plant defense against the invading pathogen.

## Introduction

*Fusarium graminearum* (telemorph: *Giberella zeae*) is a plant pathogenic ascomycete fungus causing various plant diseases on small-grain cereals such as Fusarium head blight (FHB or scrab) of wheat (*Triticium aestivum*) and barley (*Hordeum vulgare*) as well as ear and stalk rot of maize (*Zea mays*; McMullen et al., 1997; Bottalico and Perrone, 2002; Stack, 2003; Goswami and Kistler, 2004). Infection with *F. graminearum* leads to yield losses and reduction in grain quality due to shriveled, discolored kernels, or total failure of kernel development as well as to contamination with different mycotoxins, especially deoxynivalenol (DON) and zearalenone (ZEA; Trail, 2009; McMullen et al., 2012). The trichothecene mycotoxin DON is a potent inhibitor of protein biosynthesis in eukaryotes (McLaughlin et al., 1977) and causes intestinal irritation, poor weight gain and feed refusal in livestock (Eriksen and Pettersson, 2004) and may bear immunological and teratogenic effects toward human (Desjardins, 2006), whereas ZEA has estrogenic effects in human and animals (Kim et al., 2005; Gaffoor and Trail, 2006). FHB is a worldwide disease occurring among others in the United States, Canada, South America, Europe and China, whereby enormous economic losses and health threats are reported (Nganje et al., 2002).

Wheat is highly susceptible to FHB infection during the time period of anthesis (Pugh et al., 1933; Booth, 1971; Booth and Taylor, 1976; Sutton, 1982; Windels and Kommedahl, 1984; Khonga and Sutton, 1988; Trail, 2009), where temperatures ranging from 15 up to 29⋅C and high humidity represent favorable environmental conditions (Tschanz et al., 1976; Dufault et al., 2006; McMullen et al., 2012). After spore germination postulated possible entering sites for the extending fungal hyphae include wounds or natural openings like stomata, inner surfaces of palea and lemma near the floret mouth as well as crevices between the palea and the lemma (Bushnell, 2001; Lewandowski et al., 2006). The infection process is accompanied by the formation of infection cushions (Boenisch and Schäfer, 2011), an agglomeration of fungal hyphae which secrete various hydrolyzing enzymes able to degrade components of the epidermal plant cuticle and the plant cell wall, such as e.g., cutinases, pectinases, hemicellulases, cellulases, and lipases (Kang and Buchenauer, 2000; Bushnell et al., 2003; Voigt et al., 2005; Cuomo et al., 2007; Walter et al., 2010). After this initial stage of surface colonization (between the time span of roughly 20 and 70 h after infection, abbreviated hai), asymptotic intercellular fungal growth occurs, which resembles the lifestyle of biotrophic fungi. Subsequently, and from around 70 hai onwards, plant tissue necrosis occurs which is triggered by mycotoxins and intracellular growth of the pathogen (Kang and Buchenauer, 1999; Bushnell et al., 2003; Jansen et al., 2005; Boddu et al., 2006; Boenisch and Schäfer, 2011). G protein- coupled receptors have been reported to be involved in host recognition followed by downstream signaling cascades involving the mitogen-activated protein kinases (MAPK) *Fg*GPMK1 (Jenczmionka et al., 2003; Jenczmionka and Schafer, 2005) and *Fg*MGV1 (Hou et al., 2002) as well as the heterotrimeric G protein subunits Gα (*Gz*GPA1, 2, and 3), Gβ (*Gz*GPB1), Gγ (*Gz*GPG1), and the Ras-GTPase RAS2 (Bluhm et al., 2007; Yu et al., 2008; Walter et al., 2010). Finally, the classical FHB symptom of head bleaching occurs from chlorosis of the whole head. DON is necessary for this process because it allows the invading fungus to spread through the rachis from the infected to the adjacent spikelet. Through its function as translation inhibitor, DON suppresses the establishment of cell wall thickenings in the rachis node and thus inhibits this important defense response of the host (Cutler, 1988; Jansen et al., 2005; Boenisch and Schäfer, 2011). As DON induction plays such a critical role in pathogenesis its genetic regulation has been extensively studied (reviewed in Kimura et al., 2007; Walter et al., 2010). *In planta* DON production is believed to be triggered by a number of signals including specific metabolites which may pre-exist in the healthy plant or are induced in response to the pathogenic attack (such as H_2_O_2_). Some of these conditions can be mimicked *in vitro* in axenic liquid shake cultures and in fact induction of DON (and it's acetylated derivatives 15ADON and 3ADON) was found in the presence of compounds which are part of the polyamine and urea cycle pathways in plants (ornithine, arginine, agmatine, putrescine; Gardiner et al., 2009b) and/or at low pH (Gardiner et al., 2009c; Merhej et al., 2010). Polyamines and intermediates of amino acid metabolism (e.g., biogenic amines) are known to accumulate in plants attacked by pathogens (Walters, 2000, 2003) and thus it was hypothesized that *F. graminearum* might use this natural plant response to trigger DON induction (Gardiner et al., 2009b).

*F. graminearum* is among the most intensively studied fungal pathogens (Goswami and Kistler, 2004). Sequencing and annotation of its genome (Cuomo et al., 2007) as well as the development of an Affymetrix GeneChip (Güldener et al., 2006) laid the foundations of a variety of studies exploring the transcriptome of this facultative pathogen under a variety of *in vitro* growth conditions (different nutrient sources) and during different stages of infection on wheat and barley (Sieber et al., 2014).

Genome-wide expression profiles were investigated during the early developmental stages after spore germination in culture (Seong et al., 2008); under DON-inducing and non- inducing conditions in culture (Gardiner et al., 2009a); during *in vitro* growth on complete media, under nitrogen starvation and under carbon starvation. These conditions were compared with pathogenic growth during barley infection (Güldener et al., 2006) and, later on, to the transcriptome during wheat infection (Lysoe et al., 2011b). In additional surveys the transcriptome of *F. graminearum* was examined during early wheat infection (Erayman et al., 2015), during distinct infection phases of crown rot disease in wheat (Stephens et al., 2008), on dry wheat stems at different stages of colonization until perithecium formation (Guenther et al., 2009) as well as after laser capture microdissection of plant bulk material which allowed the transcriptomic examination of developmentally synchronized mycelia at distinct growth stages inside of wheat coleoptiles (Zhang et al., 2012). To test the contribution of DON production to pathogenicity-related gene expression the transcriptomes of *TRI6* and *TRI10* mutants unable to produce DON were analyzed in axenic cultures and during wheat infection (Seong et al., 2009). Developmental mutants carrying deletions in *FgStuA* were also studied in sporulation medium, on infected wheat heads as well as during secondary metabolites inducing culture conditions (Lysoe et al., 2011a). Finally, a deletion mutant in *FGP1, a* WOR-like protein was analyzed on wheat heads and under DON inducing culture conditions (Jonkers et al., 2012). All these transcriptome comparisons revealed that specific subsets of *F. graminearum* genes are exclusively expressed *in planta* and, based on these observations, were designated as “pathogenicity-related” or “virulence” genes.

It has been recognized only recently from our work and others that virulence factors such as secondary metabolites or effector proteins are not only under genetic, but also under epigenetic control in various fungi, including *F. graminearum* (Gacek and Strauss, 2012; Reyes-Dominguez et al., 2012; Connolly et al., 2013; Wiemann et al., 2013; Chujo and Scott, 2014; Soyer et al., 2014) and thus this chromatin-based regulatory mechanisms might contribute to virulence. Because we want to further study the field of epigenetic regulation in the wheat-*F. graminearum* interactions by using chromatin-modification mutants, we first sought to better understand the transcriptional response of the fungal wild-type cells to the substrate it encounters during pathogenic attack. However, some of the fungal genes expressed only *in planta* may not be related to pathogenic processes but simply responding to the specific substrate (floral tissue) while others may be directly involved in the pathogenic process (overcoming resistance). In order to better define those fungal genes which are directly associated with pathogenicity by counteracting plant defense we compared in this study *F. graminearum* transcriptomes originating from Ph-1 wild type cultures growing on living flowering wheat heads (pathogenic conditions) or on identical, but cold-killed material (saprophytic conditions). For this comparison we inoculated ears of flowering wheat heads but one half of them had been inactivated prior to infection by cutting them off the plant and dipping them into liquid nitrogen. Both samples were further incubated under the same conditions thus presenting to the fungal cells basically identical substrates but in one case under pathogenic conditions on the living “active” plant and in the other case under saprophytic conditions on the cold-killed “passive” plant material. In this paper we show that only background levels of secondary metabolites are formed on the dead plant material and that this approach is able to differentiate pathogenicity related genes from plant matrix genes and also found genes repressed by the active plant potentially revealing novel fungal targets of active plant defense.

## Results and discussion

### Experimental set up

**Figure 1:**
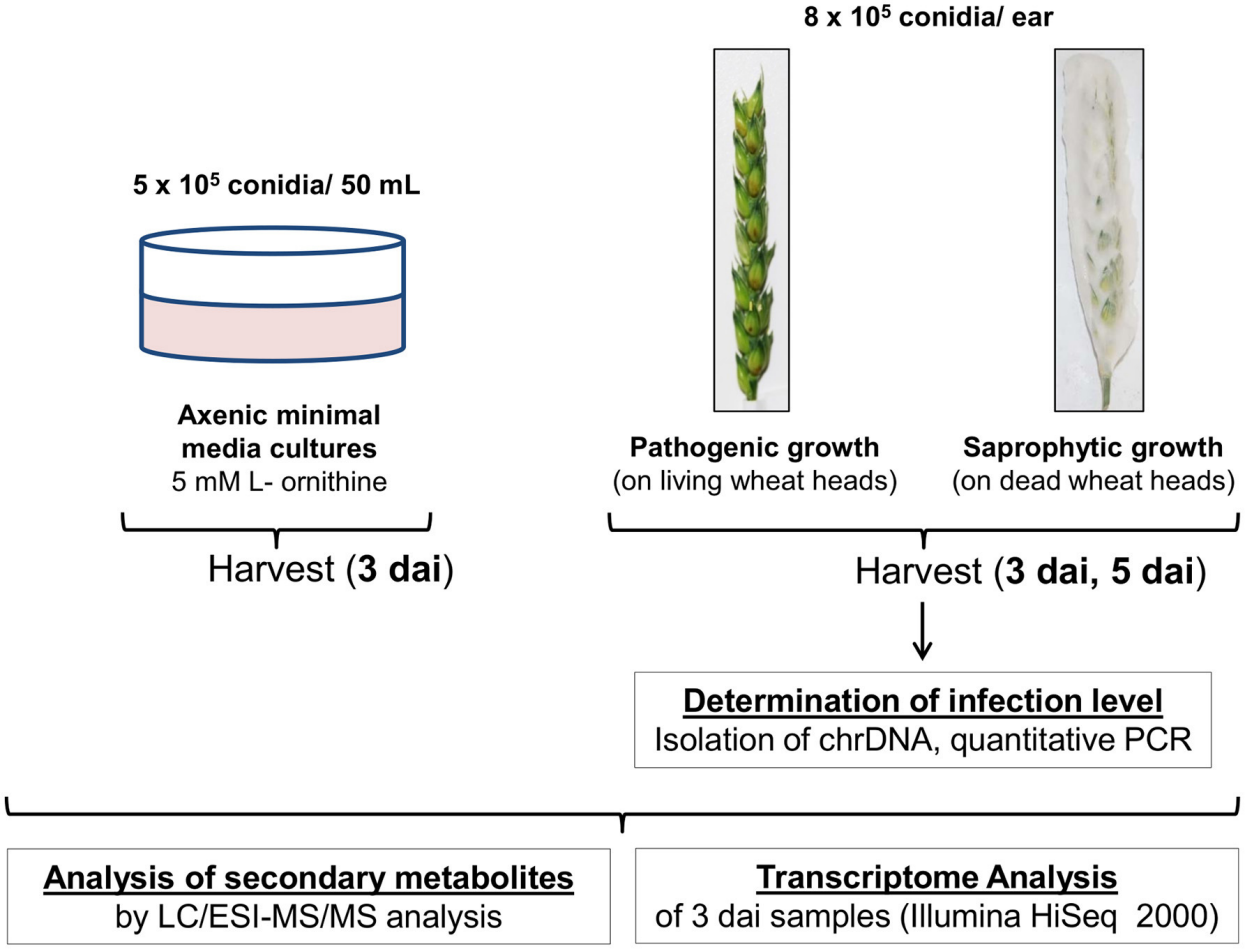
**Workflow of the experimental set up**. Three independent ears were inoculated on living plants representing pathogenic growth of the fungus. Another set of three ears, which were cut off the plant and shock-frozen in liquid nitrogen prior to spore application, was inoculated as “non- response” sample representing saprophytic growth of the fungus on the same plant material. Three and five days after inoculation (dai) samples were harvested. The infection rates were determined based on qPCR quantification of the proportion of fungal chromosomal DNA (chrDNA) within the fungus/wheat mixture (Brunner et al., 2009). Additionally to the plant experiment axenic media cultivation in presence of the DON inducing nitrogen source L-ornithine (Gardiner et al., 2009b) was carried out. All samples were subjected to chemical and molecular biological analysis. Quantitative secondary metabolite analysis was performed by LC/ESI-MS/MS against a set of analytical mycotoxin standards (Sulyok et al., 2006; Vishwanath et al., 2009). Sample processing was performed as detailed described in the text.

Figure 1 provides a schematic overview of the experimental workflow. Three independent ears were inoculated on living plants of the susceptible wheat cultivar *Remus* representing the interaction of the fungus with the living host. This condition is subsequently referred to as “pathogenic growth.” Another set of three ears, which were cut off the plant and shock-frozen in liquid nitrogen prior to spore application, was identically inoculated representing the same plant substrate but without active metabolism and defense responses. We called this condition subsequently “saprophytic growth.” The inoculated living wheat heads were cultivated under standard conditions and the inactive heads were incubated at the same location under the same conditions (see Section Materials and Methods for details). Living and dead plant material was harvested 3 and 5 days after inoculation (dai) and further analyzed for mycotoxin levels and fungal transcriptomes. In addition to these plant-based experiments we performed a standard axenic culture experiment (subsequently termed “axenic growth”) using the resting cell method (liquid minimal medium with L-ornithine as nitrogen source, growth for 3 days without shaking; see Section Materials and Methods for details).

### Active plant metabolism restricts fungal growth

**Figure 2:**
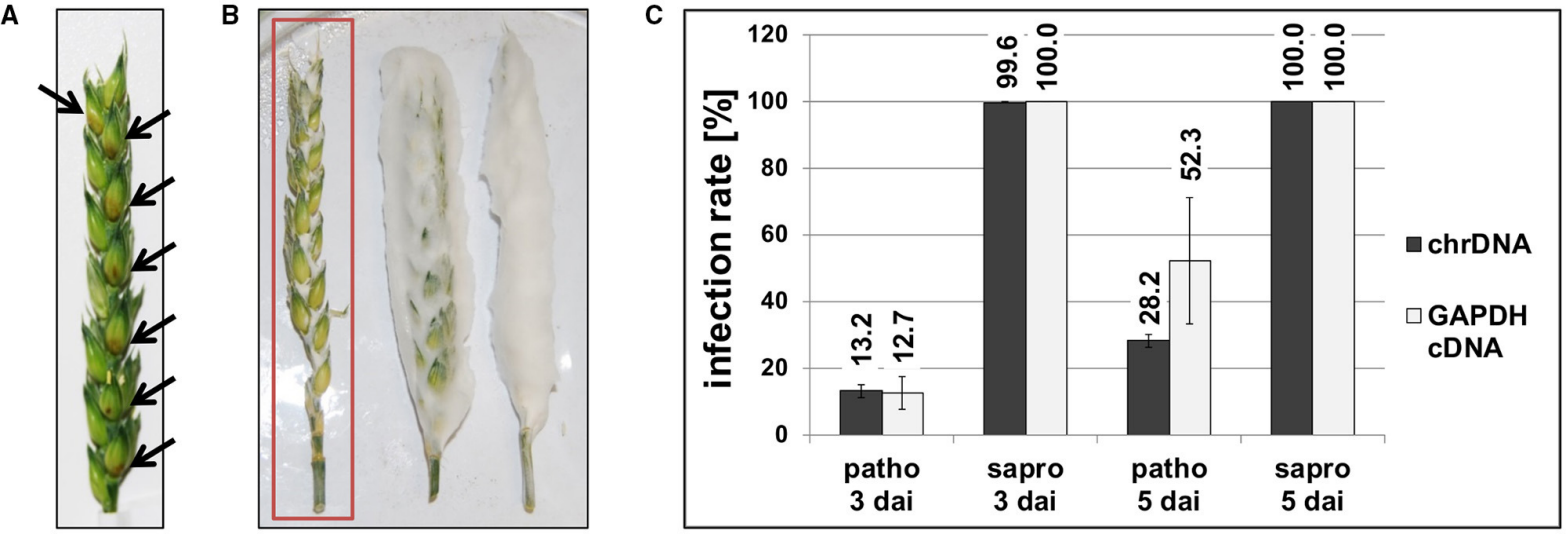
**Photographs of pathogenic (A) and saprophytic (B) ***F. graminearum*** growth on ***Remus*** wheat heads 3 days after inoculation (dai)**. Black arrows in **(A)** point toward brownish lesions visible on pathogenic samples. The red rectangle in **(B)** indicates the type of saprophytic material used for analysis. To avoid that the majority of the fungal material had no direct contact to the wheat tissue, extensive aerial hyphae were stripped off the wheat head and only the intimately connected fungal cells were used for further analysis. **(C)** Infection rates were analyzed according to the published method (Brunner et al., 2009) by DNA-based and cDNA (RNA)-based quantitative PCR. The proportion of fungal chromosomal DNA (chrDNA) was determined within the total fungal/plant DNA mixture and the proportion of fungal mRNA (GAPDH cDNA) was determined within the total fungal/plant cDNA mixture. In the saprophytic samples basically no plant-derived DNA or mRNA was detectable any more already 3 dai. Patho, pathogenic growth on living wheat heads; sapro, saprophytic growth on cold-killed wheat heads.

We first determined to which extent the fungal cells are able to proliferate on and in the wheat heads depending on whether there is active metabolism or not. Figure 2 shows example pictures of *F. graminearum* Ph-1 inoculated *Remus* wheat heads and as seen in panel A of Figure 2 a typical living wheat head 3 dai of the florets already presents clearly visible symptoms of infection (brownish lesions around the inoculation sites). Contrary to the living plant, the cold-killed wheat heads inoculated exactly the same way and incubated in the same location next to the living plant were totally overgrown by fungal mycelia. Figure 2B portrays some typical cold-killed wheat heads with extensively growing fungal mycelium around the plant material (right head). For further analysis of these saprophytically growing fungal cells we removed the portion of fluffy aerial mycelium (partial removal shown in the middle wheat head in Figure 2B) to ensure analysis of mainly plant tissue-associated fungal material (left wheat head of Figure 2B). We used qPCR to determine the relative proportion of fungal chromosomal DNA in total DNA extracted from the living or dead wheat heads using previously described methods (Brunner et al., 2009). According to this DNA-based analysis the average infection rates were 13.2% for the pathogenic and 99.6% for the saprophytic samples harvested 3 dai. After the longer incubation time (5 dai) 28.2% of the total DNA was of fungal origin in the pathogenic samples but in the saprophytic samples exclusively fungal and hardly any plant qPCR products were detected resulting in infection rates of around 100.0% (Figure 2C). This indicates that basically all plant DNA was degraded by nucleases or otherwise metabolized.

To assess, to which extent the ratio of fungal DNA to wheat DNA mirrors the activity of the fungal cells we also quantified the transcript levels of a constitutively transcribed fungal housekeeping gene relative to the transcript levels of an equivalent plant gene. Quantification of fungal and wheat GAPDH cDNA levels by qPCR (Figure 2C) gave similar results as the DNA quantifications 3 dai, i.e., 12.7% fungal GAPDH transcripts for the pathogenic and 100.0% for the saprophytic samples. Interestingly, after longer incubation (5 dai) of the pathogenic samples the relative fungal GAPDH mRNA was significantly higher (52.3%) than the equivalent DNA share (28.2%) indicating that- despite of their growth restriction by the active plant tissue- the fungal cells were highly active.

### Low mycotoxin titers during saprophytic growth on cold-killed wheat heads (passive plant tissue)

**Figure 3:**
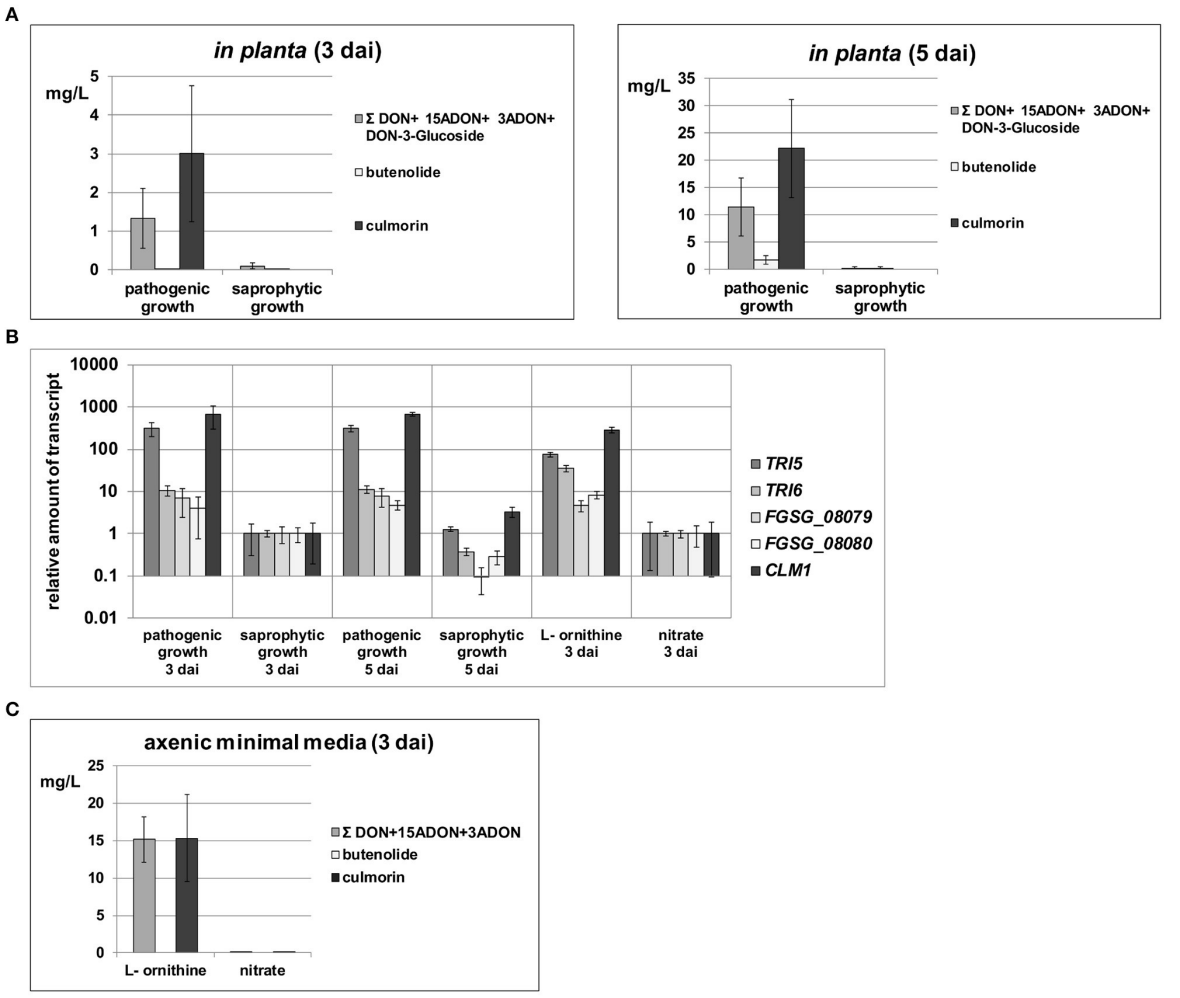
**Quantification of total DON/DON derivatives as well as butenolide and culmorin levels in different sample types and RT-qPCR analysis of key enzyme expression levels in respective SM gene clusters**. **(A)** *In planta* cell extracts were analyzed for DON and its derivatives (15ADON, 3ADON, and DON-3-Glucoside) as well as for butenolide and culmorin 3 and 5 days after inoculation (dai). **(B)** Relative transcription levels normalized to β-tubulin transcription are shown for *TRI5* (trichodiene synthase) and *TRI6* (zinc finger transcription factor) of the core trichothecene biosynthesis cluster, FGSG_08079 (predicted cytochrome P450 benzoate 4- monooxygenase) and FGSG_08080 (putative regulatory protein containing a zinc finger motif) of the butenolide synthesis cluster and *CLM1* encoding a longiborneol synthase necessary for culmorin biosynthesis. Levels were analyzed *in planta* during pathogenic (living plant) and saprophytic (dead plant) growth as well as in axenic cultures. Within the *in planta* samples expression levels of all tested genes on saprophytic material were arbitrarily set to 1 to allow direct comparison between these samples. For axenic samples, expression levels found on nitrate were arbitrarily set to 1 to allow comparisons of these *in vitro* samples. For all genes and conditions, comparative fold transcription levels are depicted on a logarithmic scale. **(C)** Filtrates of axenic cultivations on L- ornithine and nitrate harvested 3 dai were analyzed for DON and its derivatives (15ADON, 3ADON) as well as for butenolide and culmorin.

When the production of secondary metabolites was analyzed in the different samples, we found that DON (and its acetylated 15ADON and 3ADON as well as glycosylated DON-3-glucoside derivatives), butenolide and culmorin only accumulated in the living plant tissue but mycotoxin levels remained very low in the dead plant samples (Figure 3A). This is remarkable for two reasons. First, because the basic substrate for the fungal cells is identical in the living and the dead plant and second, there is much more fungal biomass accumulating during saprophytic compared to pathogenic growth. These results indicate that whatever the *in vivo* signals for mycotoxin induction are, they are not formed or unstable in the non-challenged plant cells. Obviously, for high induction of these SM biosynthetic genes the fungal invader needs host metabolites that are actively formed, most likely associated with the defense reaction, by the plant. Transcriptional analysis of the key biosynthetic genes and the transcription factors involved in the formation of the tested metabolites showed that the elevated mycotoxin levels detected during pathogenic growth on the living plant (Figure 3A) were consistent with enhanced transcription levels of these genes (Figure 3B). This demonstrates that the elevated mycotoxin levels are derived from a genetic induction event and can not only be based on biochemical changes, e.g., precursor availabilities. According to recent findings (Gardiner et al., 2010), possible candidates of such inducing plant metabolites are secondary amines (agmatine or putrescine) or intermediates of amino acid metabolism such as ornithine. It is remarkable that these metabolites are not present in the plant in sufficiently high amounts before the infection, but are produced or stabilized during the infection and are “converted” by the pathogen to a signal detrimental for the attacked host.

**Figure 4:**
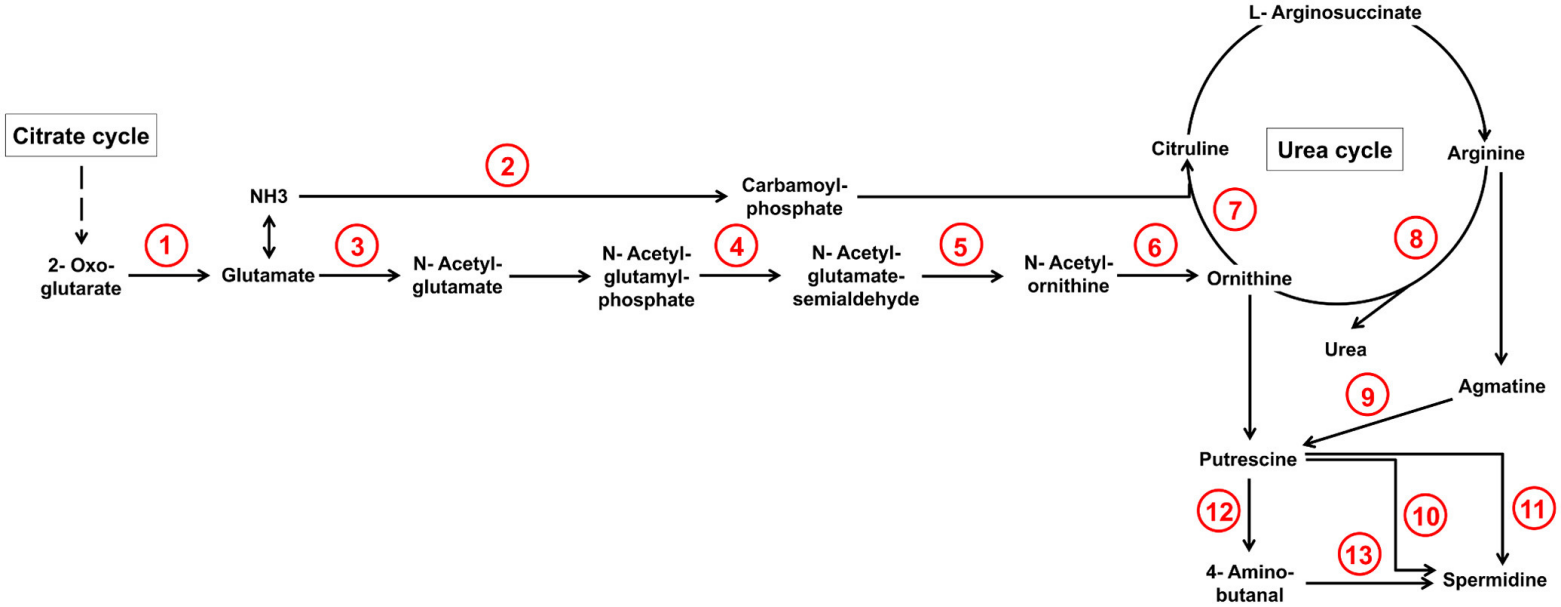
**Selection of arginine biosynthesis as well as arginine and proline metabolism reactions from KEGG database whose underlying genes showed at least a two-fold up-regulation during pathogenic in comparison to saprophytic growth**. Genes which underlie corresponding reaction numbers are shown in Table 1.

From our experimental results we can safely conclude that the concentrations of these amino acids in the dead plant material are not sufficiently high for induction and that the pathway generating ornithine or agmatine in wheat must be turned on by the fungal infection. Genes and metabolites of this pathway in fact have been identified in the combined transcriptome-metabolome studies by Nussbaumer et al. (2015). The authors found that 50 h after infection many genes involved in the polyamine/urea cycle are up-regulated in the infected wheat heads in comparison to the water-treated controls. The metabolic measurements revealed increased metabolite levels of ornithine and putrescine 96 h post infection. Our transcriptome data of the fungal cells suggest that the invading fungus reacts to these metabolic changes. We found many genes of the urea cycle and especially genes involved in the generation of ornithine and citrulline to be up-regulated (see Figure 4 and Table 1). Interestingly, citrulline was not a strong DON inducer in the study by Gardiner et al. (2009b) suggesting that the upregulation of this branch may only be necessary to provide sufficient substrate for the catabolic pathways starting from ornithine and arginine and generating the inducers ornithine, putrescine, and agmatine. Which of these mentioned metabolites act directly as inducers for the diverse SM pathways is not precisely known but based on this pathway analysis it could be that the catabolic metabolites agmatine and putrescine are the true inducers. Gene inactivation studies with these candidates would clarify this point.

**Table 1:** **Genes which are involved in arginine metabolism and were found to be at least two-fold up-regulated during pathogenic (patho) relative to saprophytic growth (sapro)**.

**FGSG number**	**Description**	***E*-value**	**Fold up- regulated (patho rel. to sapro)**	**Reaction number**
FGSG_05169	Related to aspartate aminotransferase	0	2.5	1
FGSG_11128	Probable aspartate aminotransferase, cytoplasmic	7.70E-272	3.1	1
FGSG_01573	Probable acetylornithine aminotransferase precursor	9.50E-281	2.6	1, 5
FGSG_01217	Probable carbamoyl-phosphate synthase (glutamine-hydrolyzing) arginine-specific large chain	0	3.1	2
FGSG_09638	Probable URA2—multifunctional pyrimidine biosynthesis protein	0	3.6	2
FGSG_01939	Probable amino-acid N-acetyltransferase	0	3.2	3
FGSG_06094	Probable glutamate N-acetyltransferase precursor	3.20E-282	2.0	3, 6
FGSG_05003	Probable acetylglutamate kinase/N-acetyl- gamma-glutamyl-phosphate reductase precursor (ARG-6)	1.90E-211	2.2	4
FGSG_06281	Probable ornithine carbamoyltransferase precursor	3.50E-209	2.0	7
FGSG_10967	Probable arginase	3.30E-209	3.4	8
FGSG_11721	Related to arginase	9.50E-154	2.9	8
FGSG_05446	Related to agmatinase	1.20E-254	4.7	8, 9
FGSG_03761	Related to polyamine oxidase precursor	2.60E-283	2.4	10, 13
FGSG_17334	Related to SPE-3 spermidine synthase	3.20E-192	2.1	11
FGSG_02279	Related to peroxisomal amine oxidase (copper-containing)	0	81.0	12

*E**-**values of appropriate BLAST hits are listed in the table and positions within the pathway environment of arginine metabolism are depicted schematically in Figure 4 via assigned reaction numbers*.

It is noteworthy that in the metabolomics analyses of the plant (Nussbaumer et al., 2015) ornithine, agamatine, and putrescine production was also induced by simply injecting DON into the flowering wheat heads. This demonstrates that the toxin itself triggers accumulation of these amino acids and catabolic products. For the *Fusarium* interaction this could mean that a positive feedback loop operates in which small amounts of DON produced by the initial pathogen infection induces agamatine/putrescine production which again accelerates the induction process for DON.

We also wanted to compare our *in planta* transcriptomes with axenic conditions and high DON production and consequently used 5 mM L-ornithine as sole nitrogen source. The axenic cultures were also harvested 3 dai from submerged batch cultivation. As expected, we found in these cultures high levels of DON (Figure 3C), its acetylated derivatives and culmorin (Gardiner et al., 2009b). In contrast, cultures grown on nitrate as sole nitrogen source (23.5 mM nitrogen) formed only very low levels of these metabolites (Figure 3C). L-ornithine, but not nitrate, was fully consumed during fungal biomass accumulation in these synthetic media (see Table S1) and additionally we observed a pH drop from the initial at pH 6.5 buffered media to pH 2.9 on L-ornithine whereas pH slightly rose to 6.8 on nitrate. Our data confirm published results that in axenic cultures the metabolism of L-ornithine and low pH are the responsible genetic triggers for DON induction (Gardiner et al., 2009c; Merhej et al., 2010, 2011).

### Analysis of specific gene sets responding to the different growth conditions

**Figure 5:**
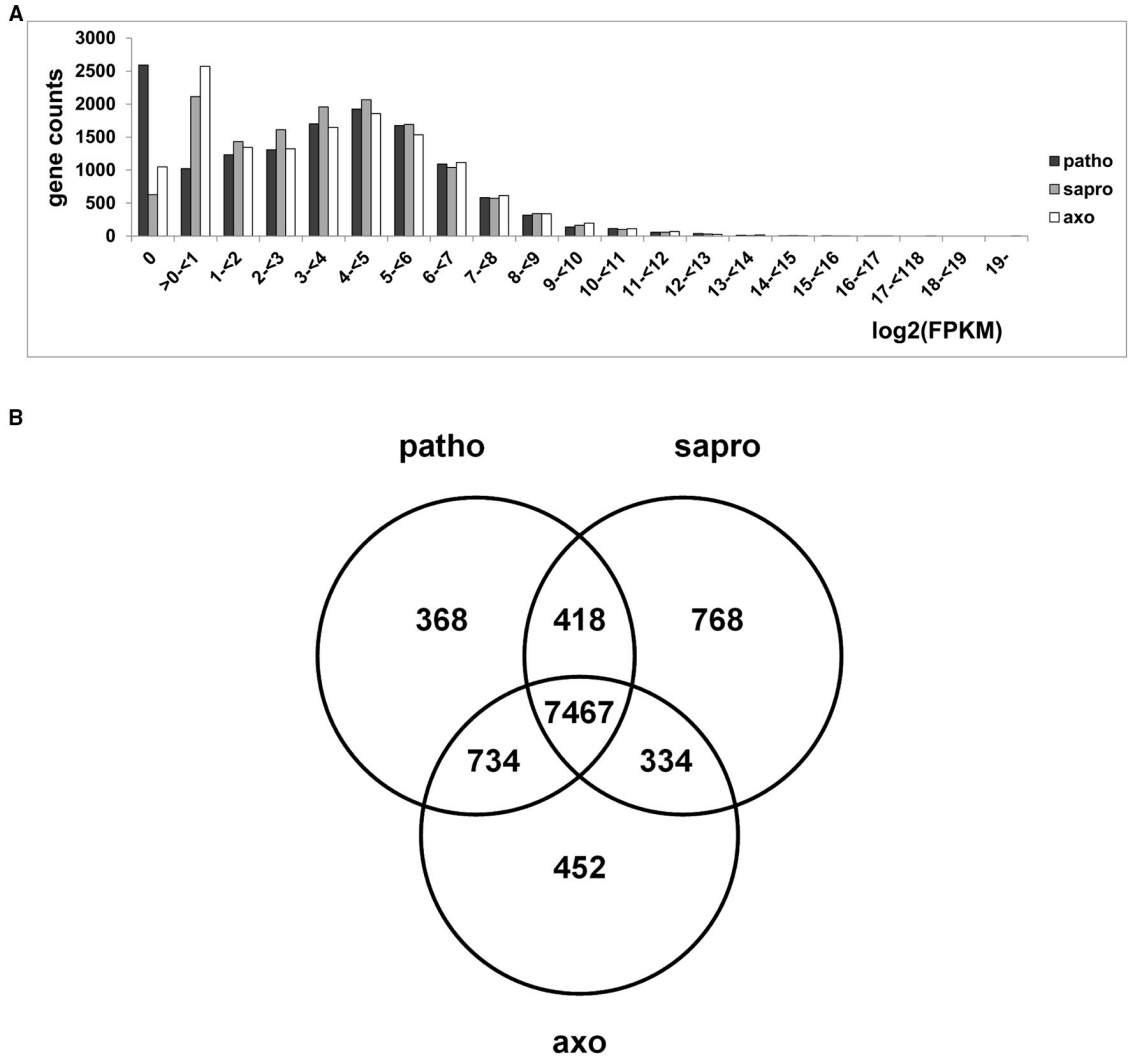
**(A)** Frequency distribution of genes counts showing certain log2(FPKM) values under the different growth conditions. **(B)** Venn diagram showing distribution of 65% highest expressed genes within the different growth conditions. Considering the proportion of 65% of highest expressed genes (8987 genes out of 13826 annotated) results in a log2(FPKM) threshold of 1.99 under pathogenic (patho), 2.46 under saprophytic (sapro), and 1.92 under axenic (axo) growth conditions.

Total RNA was isolated from axenic as well as pathogenic and saprophytic samples harvested 3 dai and all samples were independently subjected to RNA-seq analysis. Transcript levels of genes are expressed as “FPKM” values (Fragments Per Kilobase of exon per Million reads mapped) which are normalized for both sequencing depth and gene length. Figure 5A shows the number of genes in each interval of log_2_(FPKM) values for each condition. From this plot it becomes evident that under pathogenic and axenic conditions around 3500 genes of the *F. graminearum* genome are not or only rarely expressed (log_2_ < 1). Interestingly, around 1000 additional genes are expressed under saprophytic conditions. Considering the distribution of expression values over the genome, we saw that the majority of genes are expressed between ≥1 log_2_(FPKM) ≤ 5. This range covers around 9000 genes or around 2/3 of the transcribed *F. graminearum* genome. Due to these findings we considered from all predicted 13.826 genes the 65% most strongly expressed ones in each condition for further analysis. Using this cut off, all considered genes showed an expression level of at least log_2_(FPKM) ≥ 1.92 under axenic, at least log_2_(FPKM) ≥ 1.99 under pathogenic and at least log_2_(FPKM) ≥ 2.46 under saprophytic growth conditions. Relative transcript abundances in these RNA-seq data very well matched the RT-qPCR results obtained for the selected set of genes shown in Figure 3B, which were analyzed in the identical RNA extracts of all replicates and experimental conditions. The direct comparison of expression profiles of these selected five genes between RNA-seq and RT-qPCR using β-tubulin transcription for normalization of the PCR analysis is shown in Figure S1.

### Only 17% of the transcribed genome flexibly responds to conditions

Using the settings described above we found that, from all 8987 significantly expressed genes, 7467 genes were transcribed in all three samples representing 83% of the transcribed genome (Figure 5B). This means that the vast majority of genes was always transcribed, irrespectively of the greatly contrasting metabolic condition and their functions and, thus is likely associated with basic cellular processes. Overall, in our set-up, around 17% or 1528 genes were specific in their transcriptional profile for one or two of the three conditions.

The Venn diagram in Figure 5B shows that 452 genes are unique for the axenic culture conditions where growth occurs in liquid minimal medium containing sucrose as carbon and L-ornithine as nitrogen source. Consultation of the FunCat Database (https://www.helmholtz-muenchen.de/en/ibis; Ruepp et al., 2004) showed significant enrichment of functional categories such as metabolism of amino acids, and more broadly, regulation of nitrogen assimilation (see register tab “Axenic_only_452” of Table S2). This exclusivity in gene expression is most likely due to the relatively high amount of L-ornithine providing the nitrogen source in the growth medium. The second highly significant category is cellular detoxification with additional functions predicted in glutathione, glutaredoxin, and thioredoxin metabolism. This is probably due to the high mycotoxin titers found in these cells (compare to Figure 3C), but still somewhat surprising as the genes would be expected to be shared with the pathogenic conditions rather than appearing in the axenic culture category. However, the relative enrichment of genes in this gene set compared to the presence of this category in the whole genome (see register tab “Axenic_only_452” of Table S2) indicates that different gene sets are active in detoxification whether the fungus grows pathogenically or in axenic cultures.

Only few expressed genes are functionally annotated in the gene set representing the overlap between the two “non-pathogenic” conditions, i.e., axenic and saprophytic (251 unclassified proteins within the 334 genes present in this set, see register tab “Sapro_Axenic_334” of Table S2). Nitrogen regulation and amino acid metabolism is not in this commonly expressed gene set indicating that the wide variety of different nitrogen sources expected to be available to the saprophytically growing fungus is stimulating a different gene expression network compared to the exclusive L-ornithine based diet in axenic conditions.

### Individual genes within SM clusters are induced by different signals

**Figure 6:**
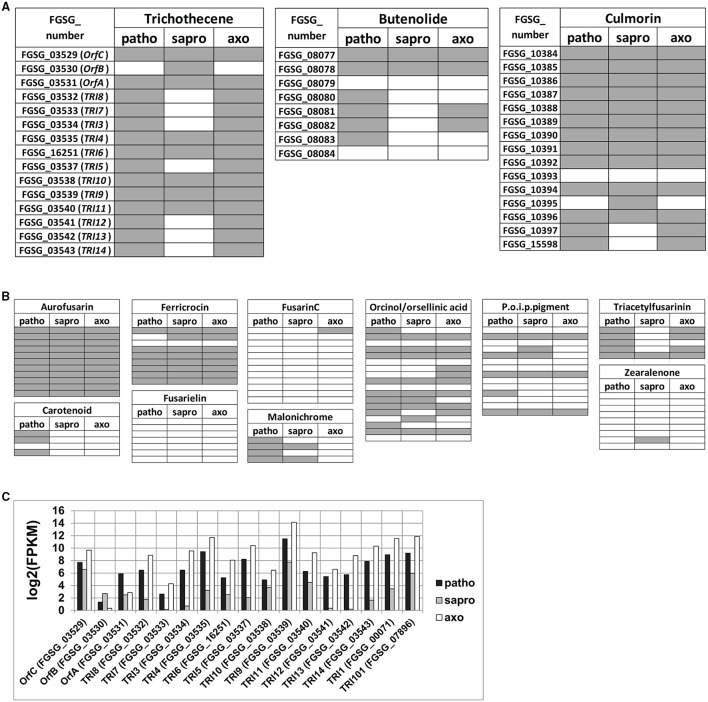
**Schematic illustration indicating which genes within secondary metabolite (SM) clusters of known products are expressed among the 65% of highest expressed genes within the respective growth condition (A,B) and detailed expression profile of the core ***TRI5*** cluster genes (***TRI8***- ***TRI14***), the three co- regulated genes (***OrfC***, ***OrfB***, ***OrfA***) as well as of the external located ***TRI1*** and ***TRI101*** genes (C)**. Horizontal fields in **(A)** and **(B)** represent one gene within the respective SM cluster. FGSG numbers (and partly gene names) are indicated for the core trichothecene, the butenolide as well as the culmorin gene clusters in **(A)**. Gray indicates expression above the log2(FPKM) thresholds demarcated by the 65% of highest expressed genes within the respective growth condition, which are 1.99 in case of pathogenic (patho), 2.46 in case of saprophytic (sapro), and 1.92 in case of axenic (axo) growth. P.o.i.p.pigment, precursor of insoluble perithecial pigment, designating cluster C53 (Sieber et al., 2014).

We used L-ornithine in the axenic cultures to exclude the known trichothecene biosynthesis genes from appearing in the gene set specific for the pathogenic growth condition. Consistent with this approach we find several genes belonging to the *TRI* cluster and coding for DON biosynthesis among the 734 gene overlap between axenic and pathogenic conditions. The same is true for genes involved in culmorin and, butenolide biosynthesis as well as for several other genes encoded within putative SM clusters involved in the biosynthesis of known as well as unknown products (Figures 6A,B and Figure S2). However, we find it intriguing that gene regulation within a given SM gene cluster does not appear to be homogenous. The *TRI* cluster expression profile under the three chosen conditions is given in detail in Figure 6C. As we found DON only under pathogenic and axenic conditions one could expect that all genes of the cluster are highly expressed in these conditions and not or weakly expressed in saprophytically grown cells. However, the thorough analysis of the cluster genes revealed a different picture. ORF-B (FGSG_03530) coding for a putative acetylesterase of unknown specificity is basically not expressed under axenic [log_2_(FPKM) = 0.3] and weakly expressed under pathogenic conditions [log_2_(FPKM) = 1.3] but strong in saprophytic samples [log_2_(FPKM) = 2.7]. This is an interesting example of a gene residing within a physically-linked gene cluster but its co-regulation is condition-dependent. In the case of ORF-B the “synthetic” *TRI* cluster inducer L-ornithine is insufficient and the plant tissues—no matter if alive or dead—must contain a compound to generate the induction signal for ORF-B.

This feature of non-homogenous gene expression is true also for many other SM gene clusters (see Figure 6B, Figure S2 and section below).

### The saprophytic gene set is dominated by plant cell wall-degrading and carbohydrate-transporting functions

Overall, there are 768 genes found to be exclusively expressed under saprophytic conditions and for 299 of these genes functions can be predicted. Not unexpectedly, around 100 of these genes exclusively expressed in the dead plant material seem to be involved in the degradation and uptake of the plant cell wall material (see register tab “Sapro_only_768” of Table S2). Functions such as enzymes for exogenous polysaccharide degradation, sugar metabolism and C-compound transport (sugar and amino acid transporters) are highly enriched functional categories in the saprophytic-specific gene set. These results suggest that a certain set of biomass degradation genes are not activated when the plant lives, i.e., in the pathogenic sample. The reason for this difference is not clear but either the specific inducer(s) for these genes are not present in the living plant or the plant defense actively prevents the transcription of these genes involved in cell wall degradation, probably by restricting the access to the substrate. However, it is surely relevant to consider for the interpretation of our saprophytic sample results that, inactivating the wheat head by dipping it into liquid nitrogen and subsequent thawing, the plant cuticles and cell walls might be partially damaged and thus easier to access by the invading fungus and its extracellular enzymes. This physical disruption might expose some substrates or liberate inducers which are not available to the fungus on the active plant.

There are also some virulence- and disease-related genes only appearing in the saprophytic sample and—similar to the cell wall-degrading enzymes—it is surprising that some of these putative pathogenicity factors are not induced when the plant defense is on. The genes code mainly for predicted membrane proteins and secondary metabolite biosynthesis of so far unknown metabolites (register tab “Sapro_only_768” of Table S2).

### Genes commonly expressed in the dead and the living plant material represent typical “*in planta*” genes

Consistent with our working hypothesis that some so-called “*in planta*” genes are not necessarily involved in overcoming the restriction posed by the defending host we found that a large portion of these genes were induced simply by the living and the dead plant tissue. The overlap between saprophytic and pathogenic conditions consists of 418 genes with many predicted functions associated with carbohydrate metabolism and transport (see register tab “Patho_Sapro_418” of Table S2).

The vast majority of genes assign to putative function in carbon compound metabolism and transport including sugar, glucoside, polyol, carboxylate, and polysaccharide metabolism. Genes coding for ion, sugar and amino acid transporters including di- and tripeptide transporters were also activated. Interestingly, the siderophore iron transport and biosynthesis system is active in dead and living plant samples although it could be hypothesized that in the saprophytic sample iron may be less limiting because the plant tissue is damaged and nutrients are exposed to the fungus. This finding indicates that the damage of the plant material by liquid nitrogen treatment was not too extensive and plant cells were not fully disrupted. On the other hand, also the pathogenically growing fungus destroys plant tissue and consistently, we found for example genes involved in vacuolar protein degradation such as alkaline proteases, aminopeptidases, and endopeptidases among the genes expressed in both pathogenic and saprophytic conditions.

Among the over-represented functional categories in this gene set were also several genes putatively involved in secondary metabolism. This is unexpected as Figure 3A shows that no classical mycotoxins are produced under saprophytic conditions however, the SM genes identified in this gene set so far cannot be assigned to a specific metabolite produced by *F. graminearum*. More broadly, secondary metabolism does not seem to be exclusive to the pathogenic condition, but also occurs in the fungus growing saprophytically or in axenic cultures. Figures 6A,B and Figure S2 give an overview of the gene distribution above the 65% threshold of highest expression within all annotated experimentally verified and predicted SM biosynthesis gene clusters (Sieber et al., 2014). The transcriptional profile of these clusters shows that the genes involved in aurofusarin production are activated in all conditions as well as seven out of nine genes within the ferricrocin cluster (Figure 6B). Some of the predicted clusters producing putative unknown metabolites or intermediates show many of their genes expressed under all tested conditions whereas for some clusters this is not the case (Figure S2). Interestingly, all ten genes of cluster C16 and all eight genes of cluster C64 are expressed under pathogenic, none of them under saprophytic and only two genes in each cluster under axenic conditions. Both clusters may be responsible for biosynthesis of unknown SMs potentially involved in pathogenicity. Generally, we find it intriguing that not all genes within a given cluster respond to the same signal and axenic conditions mimic the signals necessary for the expression of a subset of cluster genes. The molecular basis of this difference might be associated with particular promoter sequences and be relevant to better understand the living plant-specific signals leading to the production of putative virulence factors.

### The majority of genes expressed exclusively under pathogenic conditions are not associated with substrate degradation

Substrate degradation and transport activities are not enriched functional categories in pathogenic sample growing on the living plant (Figure 5B/see register tab “Patho_only_368” of Table S2). Instead we find strongly over-represented detoxification functions. In this category several multidrug resistance proteins, ABC transporters and other carriers appear in addition to enzymes putatively degrading plant defense molecules or fungal toxins produced during the infection process. This is a very clear indication that it is the active plant which triggers a fungal defense reaction and that there are basically no pre-formed or stable secondary plant or fungal metabolites which may lead to a permanent detoxification reaction in the unchallenged fungal cell.

Although we induced SM by L-ornithine in axenic cultures, there were still some specific SM genes exclusively expressed under pathogenic conditions. For example, six NRPSs with similarity to the AM-toxin forming enzymes are specific for the living plant and neither of them appear in any other sample. Thus, whereas PKS expression does not seem to be restricted to pathogenic growth, these indicated NRPS genes are. As they may code for the production of peptide toxins they might represent true virulence factors.

### 3-way comparison of differential gene expression defines the impact of the active-plant for gene expression

**Figure 7:**
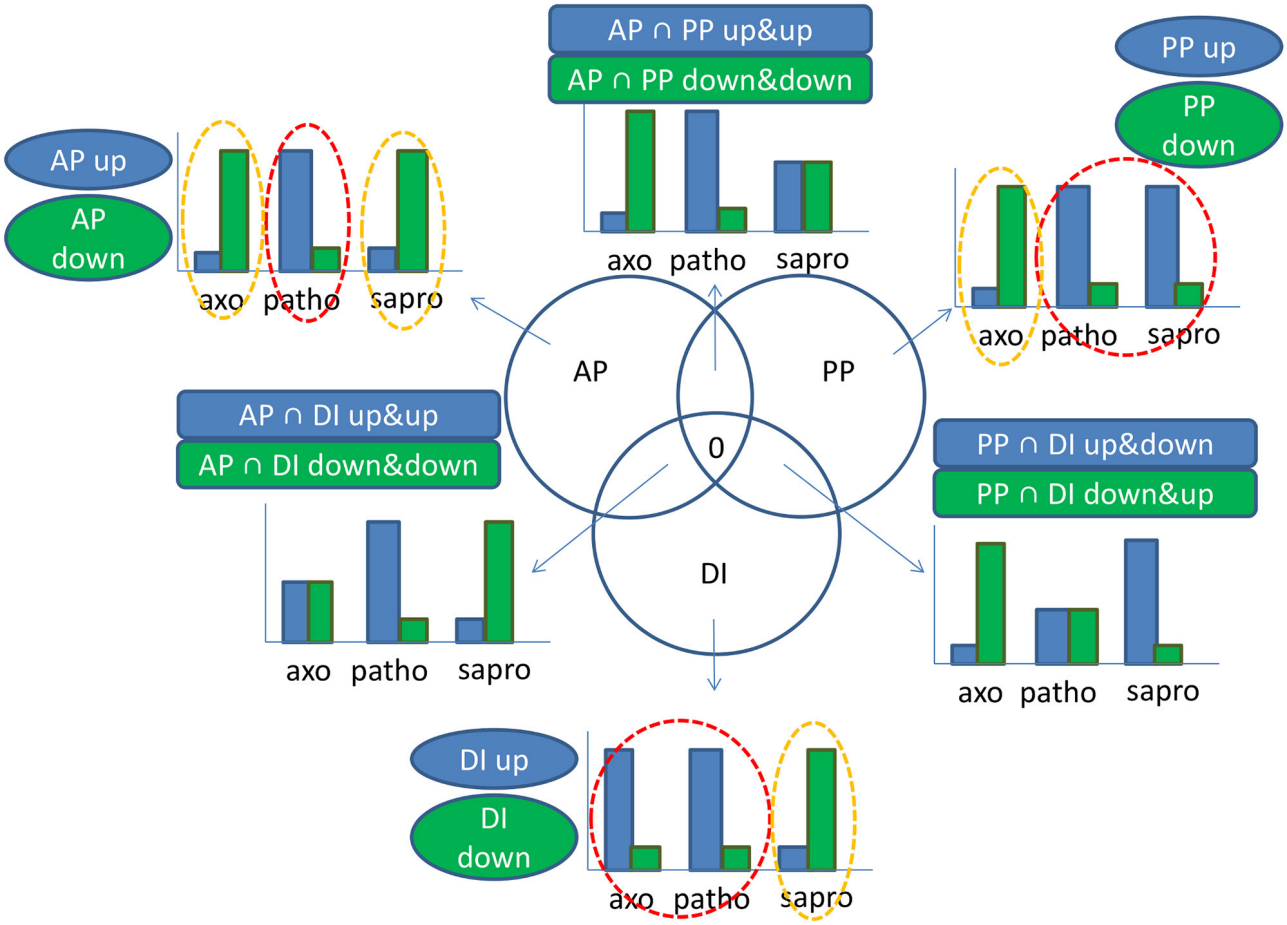
**Graphical illustration of considerations underlying the active plant (AP), passive plant (PP), and DON inducing (DI) categories which describe the main impact factors driving activation or repression of a certain gene**. In our experimental set-up we had three growth conditions: Pathogenic (patho), saprophytic (sapro), and axenic minimal media supplemented with the DON inducing nitrogen source L-ornithine (axo). Taking a minimal differential expression of four-fold between the red and orange circled conditions in account, we calculated the affiliation of a certain gene to the AP, PP, or DI categories according to following principles: In case of genes mostly regulated by the AP impact factor, transcription in the patho sample is either high and both axo and sapro are low (AP up-regulated) or transcription in the patho sample is low and both axo and sapro are high (AP down-regulated). In case of the PP impact factor we assume that plant material is present in both patho and sapro samples. PP up-regulated genes are therefore given if transcription in both patho and sapro samples is high and under axo condition transcription is low; *vice versa* PP down-regulated genes show low transcription during patho and sapro conditions but high transcription in the axo sample. Regarding DI regulated genes the patho and axo conditions have in common their DON inducing ability in addition to the limited nitrogen supply as L-ornithine was already used up in axo and intact cell walls of the defending plant restrict free nutrient access, all in contrast to the sapro condition. Therefore, genes are up-regulated by the DI impact factor if transcription in axo and patho is high but in sapro low and if transcription levels in axo and patho are low but in sapro high, genes are DI down-regulated. That means generally if two samples are similar but different from the third sample the given gene falls into one of the categories AP, PP, or DI. In case of the intersections transcription levels within all three samples are different from each other. In each case there is an intermediate level for one condition and due to this less pronounced differences in gene response we have not further considered these gene sets for further analysis. Based on these considerations a gene can exclusively fall only once in one of the specified categories. groups within the AP, PP, DI, or AP∩PP, AP∩DI, PP∩DI categories.

To gain a clearer picture on the signals specifically originating from the living plant, we performed a 3-way differential gene expression analysis considering only genes which were at least four-fold differentially regulated between the conditions. This way we were able to define the main impact factors or “drivers” of gene expression in the active plant. According to these calculations which are graphically represented in the scheme in Figure 7 we were able to define genes specifically induced or repressed by signals originating from the “active plant,” the “passive plant” or the “DON-inducing” conditions of the axenic cultures. In contrast to the list of condition-specific genes discussed above which is based on simple expression values in the three conditions this 3-way comparison of differential gene expression identifies genes which are both up-regulated or down-regulated in response to the most relevant impact a certain condition exerts on the analyzed gene. Based on this, “active plant” genes were defined as those at least four-fold higher (active plant induced) or lower (active plant repressed) expressed during pathogenic growth compared to the two other conditions. In case of these “active plant” (AP) regulated genes we assumed that they represent functions required for the pathogenic fungus to establish infections (up-regulated AP genes) or functions which may be targeted by the actively defending host and are thus down- regulated in the pathogenic fungal cells (down-regulated AP genes). In a similar reasoning we considered as “passive plant” (PP) genes those which are commonly up- or down regulated in both the pathogenic and the saprophytic samples—but differed at least four-fold from the axenic sample. Such genes are called “PP-genes” as they responded to the presence of plant tissues and metabolites, regardless if these are part of a living plant or not. This approach would also reveal only those SM genes in the AP gene sample whose induction truly requires a signal from the active plant and different to L-ornithine (present in axenic cultures). The genes differentially expressed in all three conditions fall into the overlap between AP, PP, or DI categories (see Figure 7) but as they do not respond to one specific signal, they will not be further discussed here.

**Figure 8:**
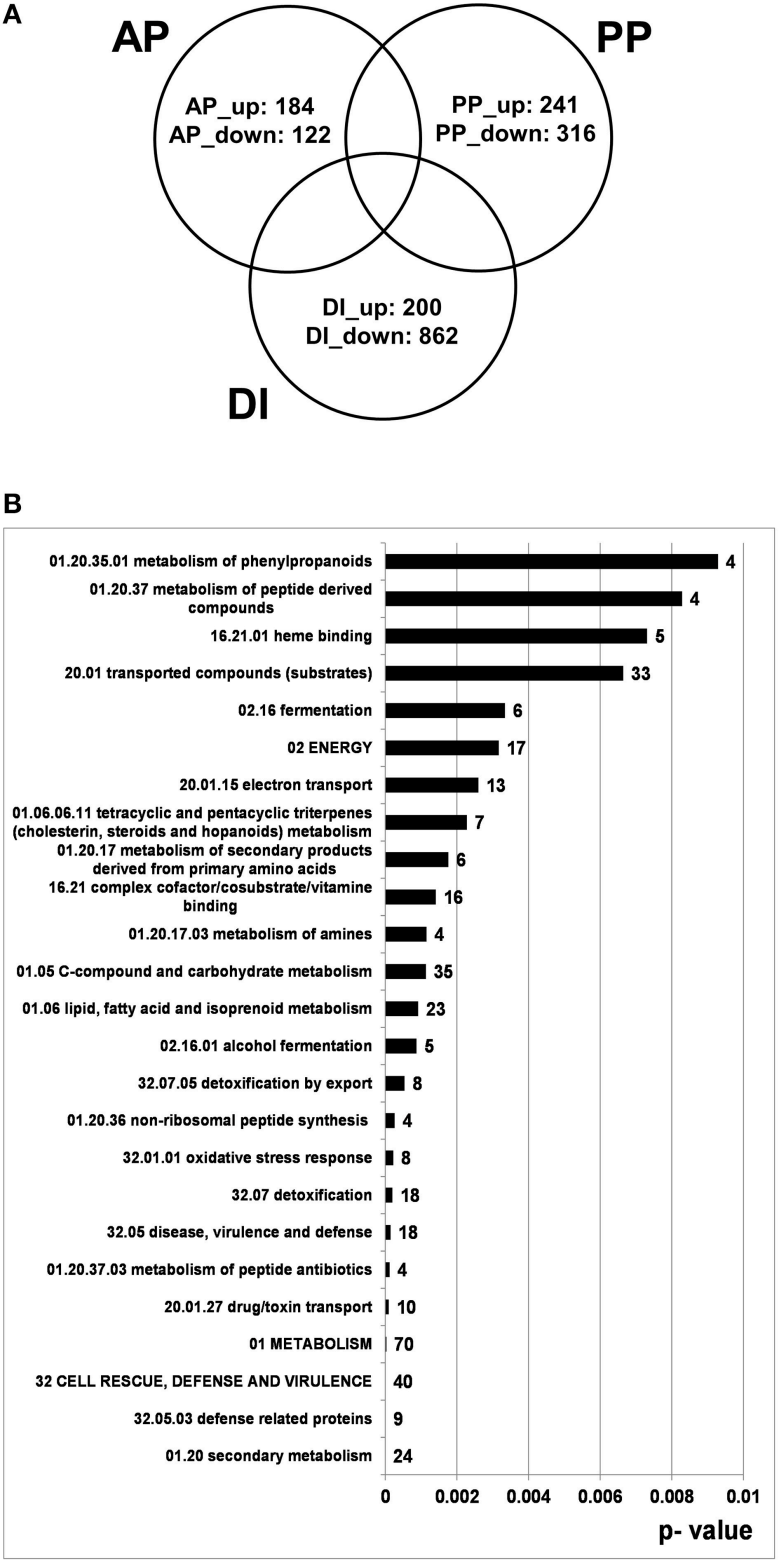
**Venn diagram showing number of genes which are at least four-fold differentially regulated between pathogenic, saprophytic and axenic L-ornithine supplemented growth conditions and could be assigned exclusively to active plant (AP), passive plant (PP), and DON inducing (DI) categories (***p*** < 0.01) (A) and FunCat analysis of 184 exclusively AP up-regulated genes (B)**. In **(A)** the appendage “_up” indicates up- and the appendage “_down” stands for down-regulation by the respective impact factor.

Our main interest was to better define the gene set specific to pathogenic conditions. By taking out genes which respond to the plant tissue (PP genes) or to the SM-inducing conditions (DI genes) we were able to restrict the large number of genes expressed under pathogenic conditions to genes with “active-plant” profiles possibly revealing novel virulence factors. According to our calculations 306 *F. graminearum* genes were specifically responding during the infection process to the living, flowering wheat heads. From this at least four-fold regulated gene set 184 genes were induced, whereas 122 genes were repressed in the AP gene set (Figure 8A). As shown in Figure 8B, functional categories most significantly overrepresented in the up-regulated active-plant gene set are associated with secondary metabolism, lipid metabolism, cellular defense and detoxification.

### Only certain known SM genes are exclusively induced by plant signals during pathogenesis

We summarized them in Table 2 and found siderophore biosynthesis genes such as one gene from the ferricrocin cluster, the NRPS, and another gene from the malonichrome as well as the NRPS gene from the triacetylfusarinin cluster. Additionally one gene involved in carotenoid formation could be classified. Apart from the two NRPS-encoding genes involved in malonichrome and triacetylfusarinin synthesis there are two other putative NRPS-encoding genes (belonging to cluster C37 and C66, respectively) which are specific for the active plant gene set but for both of them the corresponding products are not known. Furthermore, three genes of the 10-gene cluster C16 including a terpene cyclase (FGSG_04591) and four genes of the eight-gene cluster C64 including a NRPS (FGSG_10990) were classified as AP up-regulated—both clusters of which all genes were found to be expressed among the 65% of highest expressed genes during pathogenic growth (chapter “Genes commonly expressed in the dead and the living plant material represent typical ‘*in planta*’ genes”).

**Table 2:** **Putative and experimental proved secondary metabolite cluster genes classified as exclusively active plant (AP) up-regulated**.

**FGSG_number**	**Description**	SM_cluster^*^	Signature^**^	Tayloring^***^
FGSG_02279	Related to peroxisomal amine oxidase (copper-containing)	C12		
FGSG_04589	Related to tetracenomycin polyketide synthesis O-methyltransferase tcmp	C16		
FGSG_04591	Probable farnesyltranstransferase (al-3)	C16	TPC	
FGSG_04592	Related to light induced alcohol dehydrogenase Bli-4	C16		
FGSG_03956	Related to allantoate permease	C18		
FGSG_03916	Related to Rds1 protein	C19		
FGSG_02861	Uncharacterized protein	C29		
FGSG_02870	Probable ATP-binding multidrug cassette transport protein	C29		
FGSG_04693	Related to integral membrane protein PTH11	C31		
FGSG_06462	Related to coenzyme a synthetase	C37	NRPS	
FGSG_06539	Related to ACB 4-hydroxyacetophenone monooxygenase	C39		
FGSG_07582	Probable low-affinity hexose transporter HXT3	C40		
FGSG_07587	Related to sugar transport protein STL1	C40		
FGSG_13167	Uncharacterized protein	C40		
FGSG_08178	Related to decarboxylase DEC1	C48		
FGSG_10989	Related to enoyl-coa hydratase/isomerase	C64		
FGSG_10990	Related to AM-toxin synthetase (AMT)	C64	NRPS	
FGSG_10991	Related to benzoate 4-monooxygenase cytochrome P450	C64		P450
FGSG_10994	Uncharacterized protein	C64		
FGSG_11395	Related to AM-toxin synthetase (AMT)	C66	NRPS	
FGSG_11396	Related to ASN2—asparagine synthetase	C66		
FGSG_11397	Related to desaturase	C66		
FGSG_03064	Related to HSP30 heat shock protein Yro1p	Carotenoid		
FGSG_05371	Related to L-ornithine N5-hydroxylase	Ferricrocin		
FGSG_11026	Non-ribosomal peptide synthetase	Malonichrome	NRPS	
FGSG_11028	Related to ATP-binding cassette transporter protein YOR1	Malonichrome		
FGSG_03747	Related to AM-toxin synthetase (AMT)	Triacetylfusarinin	NRPS	
FGSG_03531	Monooxygenase	Trichothecene		

* *Cluster numbers/ names are according to Sieber et al. (2014)*.

** *Signature enzymes (TPC, terpene cyclase; NRPS, nonribosomal peptide synthetase)*.

*** *Tayloring enzyme (P450, cytochrome P450 monooxygenase)*.

### The AP gene set defines defense-related genes among the previously described “*in planta*” genes

In order to categorize our genes in relation to published data, we compared our AP and PP gene set defined after 72 h after inoculation to genes identified previously to be expressed specifically “*in planta*” at different time points in wheat and barley. The most comprehensive transcriptomic study taking also previous data sets into account is available from Lysoe et al. (2011b). They investigated the global gene expression of *F. graminearum* during wheat infection over time series ranging from 24, 48, 72, 96, 144, to 192 h after inoculation using microarray technology and compared their own results with published microarray data from other groups (Güldener et al., 2006). This global comparison resulted in a gene set of 591 genes exclusively expressed during infection (in wheat or both wheat and barley) whereas 9500 genes are expressed in barley, in complete or carbon or nitrogen starvation media or simultaneously during the other conditions. Importantly, fresh dead plant material like the one we used in our approach was not among the various tested substrates in the analyses carried out previously.

**Figure 9:**
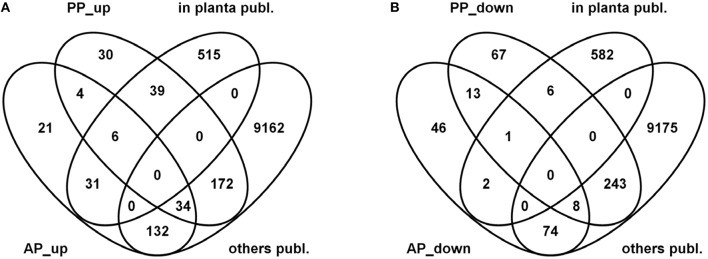
**Venn diagrams showing overlaps between active plant (AP) and passive plant (PP) up- (A) and down-regulated (B) gene sets with published datasets comprising exclusively wheat or wheat and barley induced genes (***in planta*** publ.) as well as genes expressed exclusively in barley, complete or carbon and nitrogen starvation media or combined wheat/barley/media genes (others publ.)**.

The Venn diagrams in Figure 9 show the results of this overlay between our analysis (AP, PP) and the published datasets (*in planta* publ/others publ.). For the interpretation of this comparison it is important to know that the microarray-based analysis carried out by Lysoe and coworkers considered all genes which were expressed at a certain time point regardless to which level they were induced. Thus, their plant-specific gene set contained all genes which were below a certain threshold in any of the other conditions and expressed above this threshold in infected wheat, barley or in both of them. This analysis is similar with our approach shown in Figures 5, 6A,B and Figure S2 where we considered condition-specific genes expressed above a certain threshold (contained within the 65% highest expressed gene set).

Here, we used our differential gene expression analysis which defined the AP and PP gene sets and looked for the overlap between the “plant-specific” gene set defined by Lysoe and colleagues. From the 591 “*in planta* expressed” fungal genes defined in Lysoe et al. (2011b) 31 genes could be classified as AP, 39 as PP up- regulated and 6 genes are induced regardless if the host is alive or dead whereas 515 genes do not match the up-regulated AP or PP gene sets. One important functional category not overlapping between the gene sets are genes associated with SM functions (71 genes). This difference is consistent with the use of L-ornithine as SM inducer in our axenic cultures and this leads to a removal of almost all SM-associated functions from the plant-specific gene sets. The previously published transcriptome studies, in contrast, used complete media and nitrogen or carbon starvation conditions for non-plant controls which do not, or to a much lesser extent, induce SM-associated genes and, therefore, these functions appear in their analysis in the plant-specific gene set. It is noteworthy, that also genes of the DON biosynthetic pathway, such as *TRI5*, the trichodiene synthase, or *TRI6* and *TRI10*, the two DON-cluster transcription factors, are not responding heavily to the starvation media used in these experiments and thus appear in the *in planta*-specific gene set in Lysoe et al. (2011b).

### Mainly genes responding to plant tissues are in the overlap between previously defined plant-specific genes and our up-regulated PP gene set

From the 76 genes that do overlap between previously defined *in planta*-expressed genes and our at least four-fold induced condition-specific gene sets (Figure 9) we find genes belonging to functional categories such as regulation of directional cell growth, cell wall, degradation/modification of foreign (exogenous) compounds as well as sugar, glucoside, polyol, and carboxylate metabolism (register tab “PPup_in_planta_39” of Table S3). The underlying genes are listed in Table 3 and include genes such as ones coding for a probable pectate lyase (FGSG_04430) or a cellulose binding protein (FGSG_03968). Furthermore, there are several genes predicted to encode sugar degrading enzymes, a putative monosaccharide transporter (FGSG_10921) or a probable CYB2—lactate dehydrogenase cytochrome b2 (FGSG_01531; Table 4), predicted to contribute to C-2 compound and organic acid metabolism as well as lactate fermentation (register tab “APup_PPup_in_planta_6” of Table S3). Taken together, using the described bioinformatic approach, we were here able to better describe 45 of the formerly “plant-specific” genes as activities which simply respond to the plant tissue and are thus very unlikely specific pathogenicity genes.

**Table 3:** **Thirty nine passive plant (PP) up-regulated as well as ***in planta*** expressed genes**.

**FGSG_number**	**Description**
FGSG_00096	Related to beta-galactosidase
FGSG_00540	Related to GNAT family acetyltransferase
FGSG_01570	Uncharacterized protein—related to Cutinase
FGSG_01771	Uncharacterized protein
FGSG_01829	Related to aldose 1-epimerase
FGSG_01831	Related to trihydrophobin precursor
FGSG_02914	Uncharacterized protein
FGSG_03003	Related to alpha-N-arabinofuranosidase/alpha-L-arabinofuranosidase
FGSG_03178	Uncharacterized protein
FGSG_03190	Uncharacterized protein
FGSG_03343	Related to beta-galactosidase
FGSG_03394	Uncharacterized protein—related to NPP1 domain protein
FGSG_03454	Uncharacterized protein
FGSG_03467	Probable extracellular elastinolytic metalloproteinase precursor
FGSG_03968	Related to cellulose binding protein CEL1
FGSG_04430	Probable pectate lyase 1
FGSG_04603	Related to carbonic anhydrase
FGSG_04967	Related to Cupin domain protein
FGSG_05055	Uncharacterized protein
FGSG_06463	Related to alpha-L-arabinofuranosidase A precursor
FGSG_07010	Related to stress response protein rds1p
FGSG_08093	Related to major facilitator mira
FGSG_08387	Uncharacterized protein
FGSG_08409	Related to 6-HYDROXY-D-NICOTINE OXIDASE
FGSG_09353	Related to gEgh 16 protein
FGSG_09821	Related to putative arabinase
FGSG_10411	Probable endo-1,4-beta-xylanase
FGSG_10595	Related to alkaline protease (oryzin)
FGSG_11095	Related to carbonic anhydrase
FGSG_11164	Probable trypsin precursor
FGSG_11295	Related to enoyl-CoA hydratase precursor, mitochondrial
FGSG_11302	Uncharacterized protein
FGSG_11303	Related to isotrichodermin C-15 hydroxylase (cytochrome P-450 monooxygenase CYP65A1)
FGSG_11305	Related to CSR1—phosphatidylinositol transfer protein
FGSG_11315	Uncharacterized protein
FGSG_11367	Related to lactose permease
FGSG_16622	Uncharacterized protein
FGSG_17574	Non-ribosomal peptide synthetase
FGSG_17621	Uncharacterized protein

**Table 4:** **Six active and passive plant (AP and PP) up-regulated as well as ***in planta*** expressed genes**.

**FGSG_number**	**Description**
FGSG_00028	Probable metalloprotease MEP1
FGSG_01468	Uncharacterized protein
FGSG_01531	Probable CYB2—lactate dehydrogenase cytochrome b2
FGSG_05296	Uncharacterized protein
FGSG_10921	Related to monosaccharide transporter
FGSG_11027	Related to N6-hydroxylysine acetyl transferase

Additionally, we could identify within our experiment 30 new genes within the PP category which had not been found previously to be plant-specifically expressed (Table 5). The large proportion of genes with predicted functions among them act in cellular transport contributing to cellular import, carbohydrate transport, ion transport and homeostasis of cations and phosphate as well as peroxisomal transport (register tab “PPup_30” of Table S3).

**Table 5:** **Thirty new passive plant (PP) up-regulated genes**.

**FGSG_number**	**Description**
FGSG_00840	Probable carnitine acetyl transferase FacC
FGSG_00885	Uncharacterized protein
FGSG_02070	Related to putative tartrate transporter
FGSG_02076	Uncharacterized protein
FGSG_03589	Related to 4-coumarate–CoA ligase
FGSG_04288	Related to 26S proteasome subunit RPN4
FGSG_04390	Uncharacterized protein
FGSG_04599	Related to peroxisomal short-chain alcohol dehydrogenase
FGSG_06545	Related to aminopeptidase Y precursor, vacuolar
FGSG_07026	Uncharacterized protein
FGSG_09191	Related to formate transport protein
FGSG_11154	Uncharacterized protein
FGSG_11648	Uncharacterized protein
FGSG_12485	Related to ferric reductase Fre2p
FGSG_13227	Uncharacterized protein
FGSG_13392	Uncharacterized protein
FGSG_13438	Uncharacterized protein
FGSG_13761	Uncharacterized protein
FGSG_13834	Related to bromodomain protein bdf1
FGSG_15187	Uncharacterized protein
FGSG_15274	Uncharacterized protein
FGSG_15790	Related to putative multidrug transporter
FGSG_15864	Related to PHO-4 phosphate-repressible phosphate permease
FGSG_16195	Related to methyltransferase
FGSG_16210	Uncharacterized protein
FGSG_16329	Related to ANX-14 annexin XIV
FGSG_16340	Related to phytoene dehydrogenase AL-1 (carotenoid biosynthesis protein al-1)
FGSG_16683	Uncharacterized protein
FGSG_16878	Probable glucokinase
FGSG_17076	Related to phenol 2-monooxygenase

### Many up-regulated genes in the PP category code for secreted proteins

**Figure 10:**
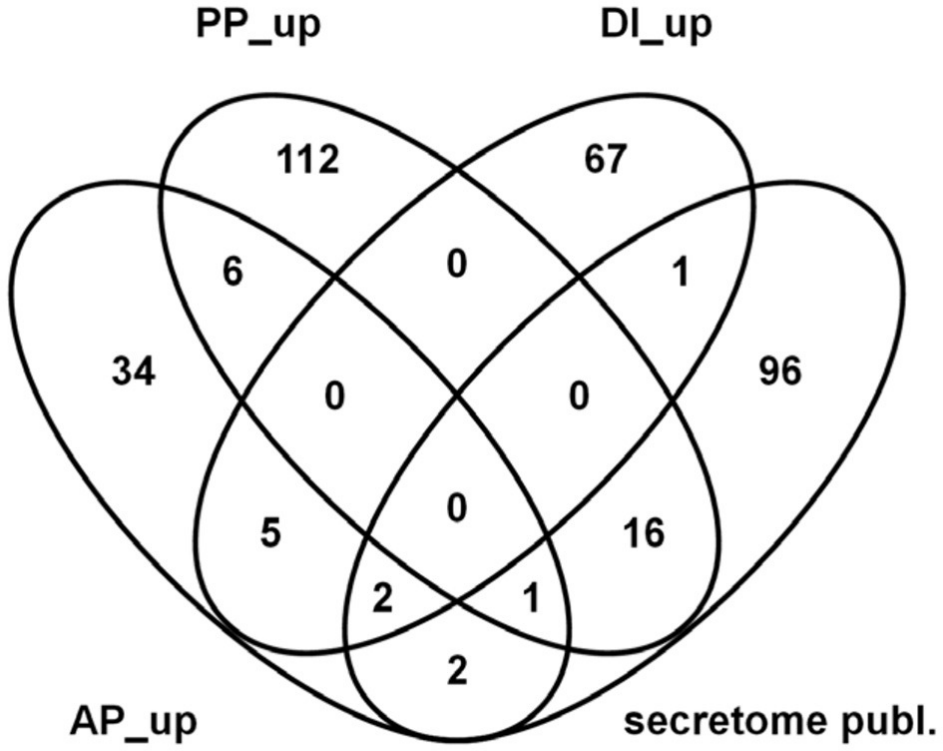
**Venn diagram showing overlaps between published ***in planta*** secreted proteins (Paper et al., 2007) and predicted genes coding for secreted proteins in the actual dataset which are up- regulated in the active plant (AP), passive plant (PP) and/or DON inducing (DI) categories**.

Many of our PP-specific genes code for proteins which carry secretion signals and are thus likely to be externalized during growth. Consequently, we also compared this gene set to the proteins found in a proteomic study to occur in apoplastic fluids of infected wheat (Paper et al., 2007). From the 120 proteins previously identified in the proteomic study we found 118 genes in our experimental set up and by considering only genes that encode predicted secretion proteins we could identify 16 genes to be PP up-regulated and one gene to be positively regulated by AP and PP impact factors (see Figure 10). The PP up-regulated functional categories comprise genes coding for cytoplasmic, nuclear, lysosomal, and vacuolar protein degradation as well as for proteolytic protein processing and extracellular polysaccharide degradation. Examples are proteins similar to secreted alpha-N-arabinofuranosidase/alpha-L-arabinofuranosidase (FGSG_03003), to vacuolar aminopeptidase Y precursor (FGSG_03027), to endo-1,4-beta-xylanase A precursor (FGSG_03624) and a predicted cellulase (FGSG_11184). The one gene that is positively affected by AP and PP impact factors, FGSG_00028, is annotated as probable metalloprotease MEP1 likely to be involved in protein/peptide degradation.

### One part of the AP gene set overlaps with known “*in planta*” genes and contains many SM precursor functions

The 31 AP genes which overlap with the previously identified *in planta* genes show functional enrichment in categories of tyrosine, phenylpropanoid, and triterpene metabolism as well as metabolism of peptide antibiotics, defense-related proteins and secondary metabolism (register tab “APup_in_planta_31” of Table S3 and Table 6). The genes have been previously identified as “*in planta*” expressed but it is interesting that the genes with SM-related functions appear in our analysis not together with the classical SM genes such as DON or culmorin cluster genes. This indicates that—among the known SM genes—there are genes which cannot be induced by the pathway described above involving L-ornithine as inducer compound. This seems to be true for the farnesyltransferase involved in the provision of general precursor farnesylpyrophosphate (FPP) and the tyrosinase which is part of the shikimate pathway important for SM precursors. Among the “defense-related” functions there are the two AM toxin genes and the third one, FGSG_11542 most likely encodes an aldehyde dehydrogenase involved in acetate formation. It thus may also be part of active-plant responsive SM biosynthesis as acetate can serve as a building block for several SMs. Induction of all these SM-related functions is clearly dependent on the actively defending plant and it would be worthwhile to study gene regulation of this gene set to understand the difference between the “classical” SM genes induced by the ornithine pathway also in axenic cultures and these genes which are strictly dependent on the active plant.

**Table 6:** **Thirty one active plant (AP) up-regulated as well as ***in planta*** expressed genes**.

**FGSG_number**	**Description**
FGSG_00237	Related to trichothecene 3-O-acetyltransferase
FGSG_02432	Related to formaldehyde dehydrogenase
FGSG_03175	Related to quinone reductase
FGSG_03531	Monooxygenase
FGSG_03916	Related to Rds1 protein
FGSG_04589	Related to tetracenomycin polyketide synthesis O-methyltransferase tcmp
FGSG_04591	Probable farnesyltranstransferase (al-3)
FGSG_04592	Related to light induced alcohol dehydrogenase BLI-4
FGSG_04615	Uncharacterized protein
FGSG_04773	Related to endoglucanase IV precursor
FGSG_07612	Related to general amidase
FGSG_07807	Uncharacterized protein
FGSG_08178	Related to decarboxylase DEC1
FGSG_08193	Related to DUF1237 domain protein
FGSG_08765	Uncharacterized protein
FGSG_09291	Probable pectate lyase 1
FGSG_10081	Related to hard surface induced protein 3 (chip3)
FGSG_10989	Related to enoyl-CoA hydratase/isomerase
FGSG_10990	Related to AM-toxin synthetase (AMT)
FGSG_10991	Related to benzoate 4-monooxygenase cytochrome P450
FGSG_11028	Related to atp-binding cassette transporter protein YOR1
FGSG_11046	Uncharacterized protein
FGSG_11047	Uncharacterized protein
FGSG_11106	Related to monophenol monooxygenase (tyrosinase)
FGSG_11123	Related to N-acetylgalactosamine-6-sulfatase precursor
FGSG_11395	Related to AM-toxin synthetase (AMT)
FGSG_11396	Related to ASN2—asparagine synthetase
FGSG_11397	Related to desaturase
FGSG_11399	Related to oxidoreductase
FGSG_11542	Related to aldehyde dehydrogenase (NAD), mitochondrial
FGSG_15448	Uncharacterized protein

### Expression pattern of known pathogenicity factors in our data set

In their great review Walter et al. (2010) published a table that shows pathogenicity and virulence factors produced by *F. graminearum* and *F. culmorum* which are known in literature to reduce or even lead to loss of virulence when mutated. The trichodiene synthase-encoding gene *TRI5* (Proctor et al., 1995a, 1997; Desjardins et al., 2000) and the transcription factor *TRI6* (Proctor et al., 1995b; Hohn et al., 1999) are well-known examples, which showed the highest transcription during axenic growth in our experiment and could be assigned to the DI up-regulated (*TRI5)* and PP down-regulated (*TRI6*) impact factor categories. The extracellular secreted lipase-encoding gene *FgFGL1* (Voigt et al., 2005) was strongest transcribed in the saprophytic samples, whereas *FgNPS6* (Oide et al., 2006) and *FgSID1* (Greenshields et al., 2007) both involved in iron metabolism showed the highest transcription during infection and both could be classified as AP up-regulated genes. Furthermore, the *F. graminearum* homolog of *FcABC1* (Skov et al., 2004), encoding a PDR ABC transporter possibly involved in transport of phytoanticipins, and *GzARG2* (Kim et al., 2007), encoding an acetylglutamate synthase involved in arginine biosynthesis both showed highest transcription during pathogenic growth and *Fg*/*FcABC1* could be assigned as AP up-regulated, too. The other listed pathogenicity factors showed a similar transcription in all three sample types, which does not mean that they are not important for the infection process (since their mutation causes reduced virulence) but might indicate, that their transcriptional regulation does not necessarily need signals from the active, living plant to be switched on. Additionally posttranscriptional regulation might take place modifying or fine-tuning activity of the gene product. Cuomo et al. (2007) published a list of possible virulence factors among predicted secreted proteins that were specifically expressed *in planta* and found in high SNP density regions within the fungal genome. The list contains putative homologs of known virulence factors, cell wall-degrading enzymes and cytochrome P450s. Most of the cell wall-degrading enzymes show similar transcription levels on the pathogenic and the saprophytic samples, some a higher level on the latter one. The pectate lyase FGSG_03483, however, in addition to the secreted protease FGSG_00028 and the cytochrome P450 oxidase FGSG_10991, show significant higher induction during infection of the living plant compared to saprophytic and axenic growth and, thus, may represent interesting candidate genes for further study (out of this three FGSG_10991 could be classified as AP up-regulated). In our experimental setup we could dissect three factors that impact on the invading fungus and vary its transcriptional response accordingly. Targeting genes that are affected by the active plant response may be a promising strategy for countermeasures against this fungal disease.

### The AP category also contains putatively novel virulence factors

The most interesting category in our analysis is surely the 21 genes in the AP category which have not yet been considered in any of the previous studies. As enriched functional categories we found here disease, virulence and defense as well as detoxification, drug/toxin transport, and detoxification by export (register tab “APup_21” of Table S3) comprising genes such as FGSG_02672 (probable cytochrome P450 monooxygenase (lovA)-encoding gene), FGSG_03080 (related to ethionine resistance protein), FGSG_00101 (related to integral membrane protein), FGSG_16401 (probable ATP-binding cassette multidrug transport protein ATRC; Table 7). Isoprenoid and terpene metabolism was also enriched additionally including FGSG_09863 (related to acyl-CoA cholesterol acyltransferase). Furthermore, the gene set includes FGSG_02917 (related to cellobiose dehydrogenase) and FGSG_04057 (probable potassium transporter TRK-1) as well as FGSG_02263 (uncharacterized protein—related to ABC-type Fe3 transport system, periplasmic component) and FGSG_03186 (related to oxidoreductase).

**Table 7:** **Twenty one new active plant (AP) up-regulated genes**.

**FGSG_number**	**Description**
FGSG_00101	Related to integral membrane protein
FGSG_02263	Uncharacterized protein—related to ABC-type Fe3 transport system, periplasmic component
FGSG_02672	Probable cytochrome P450 monooxygenase (lovA)
FGSG_02917	Related to cellobiose dehydrogenase
FGSG_03080	Related to ethionine resistance protein
FGSG_03186	Related to oxidoreductase
FGSG_03640	Uncharacterized protein
FGSG_04057	Probable potassium transporter TRK-1
FGSG_06913	Uncharacterized protein
FGSG_09863	Related to acyl-CoA cholesterol acyltransferase
FGSG_10241	Uncharacterized protein
FGSG_10994	Uncharacterized protein
FGSG_11101	Uncharacterized protein
FGSG_13383	Uncharacterized protein
FGSG_13536	Related to PRM10 pheromone-regulated protein
FGSG_16218	Uncharacterized protein
FGSG_16401	Probable ATP-binding cassette multidrug transport protein ATRC
FGSG_16849	Related to seed maturation protein pm25
FGSG_16872	Uncharacterized protein
FGSG_16938	Uncharacterized protein
FGSG_17049	Uncharacterized protein

Novel SM-related genes in this category feature an acyl-coA transferase with putative functions in sterol metabolism (FGSG_09863). This type of acyltransferases are known to be membrane-bound proteins that utilize long-chain fatty acid acyl-CoA and sterols as substrates to form steryl esters as precursors of ergosterol biosynthesis (Madhosingh and Orr, 1981; Perkowski et al., 2008; Breakspear et al., 2011). The P450 monooxygenase-encoding gene (FGSG_02672) is not part of any established SM cluster and may thus, have different non-secondary metabolic functions. Several transporters are part of this AP gene list including predicted iron and potassium transporters, multidrug resistance proteins as well as an efflux pump for the methionine analog ethionine (FGSG_03080). The protein may be involved in methionine-related C1 metabolism, most likely *via* connection to SAM (S-adenosylmethionine) metabolism which was shown to be affected in ethionine-resistant mutants (Barra et al., 1996). An interesting gene is represented by FGSG_02917, which might encode an extracellular protein of the haemoflavoenzyme family because proteins with significant similarity are able to convert cellobiose and other cellodextroses to their respective lactones (Henriksson et al., 2000). This function of the AP-induced gene might hence be necessary to induce further plant tissue-degrading enzymes. Another yet unrecognized gene with a putative virulence function might be represented by FGSG_00101 which encodes a protein similar to Pth11 from *Magnaporthe oryzae*, a GPCR localized to membranes and required for appressorium differentiation and, thus, pathogenesis (DeZwaan et al., 1999; Lu et al., 2005).

Until recently, not much was known about small secreted cysteine-rich proteins (SSCPs) or “effectors” in *F. graminearum*. This secreted protein family plays a prominent role in other pathosystems including *Fusarium* species (de Sain and Rep, 2015). A recent genomic analysis, however, predicted 190 candidate genes which conform to the characteristics of SSCPs with a possible effector function (Lu and Edwards, 2016). In this study, 25 out of the 190 predicted peptides were confirmed by proteomics in axenic media culture supernatants and 23 of these were found by semi-quantitative RT-PCR to be expressed to different degrees *in planta* during the infection process. We queried our dataset for the expression levels of these possible effectors and found 48 of the predicted genes in the AP, PP, and DI categories to be differentially expressed more than four-fold (Table 8). Only six of the confirmed 23 SSCPs appeared to be strongly regulated in our comparison. Three of them (FGSG_04805, FGSG_05714 and FGSG_11205) were strongly down-regulated by the active plant response (category AP_down) and, thus, may be targets of the plant defense system. Two were found to be up-regulated under DON-inducing conditions (DI-up, i.e., high in pathogenic and axenic conditions) indicating that they are co-regulated with DON and, thus, be part of the regulatory circuit for virulence factor expression. Finally, the last confirmed SSCP-encoding gene in our dataset is FGSG_13952, which assigns to the category of down-regulated genes in the DON-inducing conditions. Thus, this secreted peptide seems to be inversely regulated compared to DON, very highly expressed under saprophytic conditions, most likely induced by a dead plant matrix component.

**Table 8:** **Out of the 190 small secreted cysteine-rich proteins (SSCPs) identified in the genome of ***F. graminearum*** (isolate PH-1) by Lu and Edwards (2016), we could assign 48 exclusively to active plant (AP), passive plant (PP), or DON- inducing (DI) up- or down-regulated categories within our analysis (indicated by gray colored fields)**.

**FGSG number**	**Description**	**AP**		**PP**		**DI**	
		**Up**	**Down**	**Up**	**Down**	**Up**	**Down**
FGSG_00056	Uncharacterized protein						
FGSG_00060^**^	Related to KP4 killer toxin						
FGSG_00061^**^	Related to KP4 killer toxin						
FGSG_00062^**^	Related to KP4 killer toxin						
FGSG_00114	Uncharacterized protein						
FGSG_00230	Uncharacterized protein						
FGSG_01239	Uncharacterized protein						
FGSG_01771	Uncharacterized protein						
FGSG_01831^**^	Related to trihydrophobin precursor						
FGSG_02685	Uncharacterized protein						
FGSG_02935	Uncharacterized protein						
FGSG_03130	Uncharacterized protein						
FGSG_03157	Uncharacterized protein						
FGSG_03326	Uncharacterized protein						
FGSG_03599^**^	Uncharacterized protein						
FGSG_03894	Uncharacterized protein						
FGSG_03911	Uncharacterized protein						
FGSG_03960	Uncharacterized protein						
FGSG_04661^**^	Uncharacterized protein						
FGSG_04745	Related to antifungal protein						
FGSG_04805^**^	Uncharacterized protein						
FGSG_05714^*^	Uncharacterized protein						
FGSG_07807	Uncharacterized protein						
FGSG_07972	Uncharacterized protein						
FGSG_08085	Uncharacterized protein						
FGSG_08122^*^	Uncharacterized protein—related to cell wall protein phia						
FGSG_08142	Uncharacterized protein						
FGSG_08164^*^	Uncharacterized protein						
FGSG_09066	Uncharacterized protein						
FGSG_09133	Uncharacterized protein						
FGSG_09403	Uncharacterized protein						
FGSG_10554	Uncharacterized protein						
FGSG_11047	Uncharacterized protein						
FGSG_11205^*^	Probable snodprot1 precursor						
FGSG_11225	Uncharacterized protein						
FGSG_11540	Uncharacterized protein						
FGSG_11564	Uncharacterized protein						
FGSG_12554	Uncharacterized protein						
FGSG_12673	Uncharacterized protein						
FGSG_12722	Uncharacterized protein						
FGSG_12981	Uncharacterized protein						
FGSG_13412	Uncharacterized protein						
FGSG_13462	Uncharacterized protein						
FGSG_13464	Uncharacterized protein						
FGSG_13491	Uncharacterized protein						
FGSG_13505	Uncharacterized protein						
FGSG_13952^**^	Uncharacterized protein						
FGSG_14010	Uncharacterized protein						

Of these, 12 genes are among the 34 SSCPs detected by Lu and Edwards (2016) during transcriptional analysis of infected wheat heads- FGSG numbers marked by one asterisk (^*^). FGSG numbers marked by two asterisks (^**^) are among the 15 in planta SSCPs with transcripts up-regulated at certain point. Underlined FGSG numbers are among the 25 SSCPs confirmed by nanoscale liquid chromatography-tandem mass spectrometry (nanoLC-MS/MS) analysis of the minimal medium-based in vitro secretome.

Interestingly, there are four genes of the 190 predicted SSCPs which assign to our AP_up category because they are highly induced only in the actively defending plant (FGSG_01239, FGSG_07807, FGSG_11047, and FGSG_12554). Unfortunately, the corresponding peptides have not been found in the synthetic media secretome and thus, they are not confirmed SSCP-encoding genes. However, the fact that they are highly induced only in the active plant indicates that they might have a signaling or regulatory function during the *F. graminearum*-wheat interaction.

### Genes repressed in the AP gene set might represent important fungal targets of plant defense

So far we have mainly discussed genes up-regulated by the signals derived from the actively defending plant. But also genes specifically repressed by the active plant are interesting as they may represent possible targets of the plant defense system to keep the pathogen under control.

In this category (Figure 9B) we identified 46 genes repressed by signals originating from the active plant and which were not found by Lysoe et al. (2011b). Unfortunately, the vast majority in this gene set (42 genes) encodes putative proteins of unknown function whereby notable four genes in this set are predicted to encode short peptides with a predicted amino acid (AA) length between 100 and 210 AAs and a cysteine content above 3%: FGSG_00860 (209 AA, 6.7% cys. content), FGSG_12722 (143 AA, 5.6% cys. content), FGSG_13097 (105 AA, 3.8% cys. content), and FGSG_13598 (103 AA, 4.9% cys. content). Thus, they may represent small cysteine-rich effectors which may play a role in pathogen recognition or other processes and the fact that they are actively repressed by the living plant signals is certainly an interesting observation. If these peptides contribute to virulence, however, remains to be shown in future studies. The remaining four genes with predicted functions are listed in Table 9.

**Table 9:** **Only four out of 46 new AP down-regulated genes show a predicted gene function**.

**FGSG_number**	**Description**
FGSG_02115	Related to TRI7—trichothecene biosynthesis gene cluster
FGSG_02259	Related to ATP adenylyltransferase II
FGSG_15857	Anaphase promoting complex subunit 11
FGSG_15891	Related to *trans*-aconitate 3-methyltransferase

## Conclusions

The aim of our study was to better define the group of “*in planta*” expressed fungal genes because the pathogenic growth status represents a mixture of genes responding to the plant tissue as substrate already present (such as cell wall compounds and pre- formed metabolites) as well as genes responding to defense signals and metabolites built up in response to the pathogenic attack. This genetic differentiation is surely relevant because it has the potential to identify novel pathogenicity factors. In addition, potentially novel plant defense targets may be present in the set of down-regulated fungal genes identified uniquely in the living plant. Our approach has led to a differentiation of genes responding to these mixed life styles and we identified 184 genes upregulated and 122 genes repressed at least four-fold by the signals the active plant generates during the infection process. We found in this gene set a substantial overlap with previously characterized “*in planta*” expressed genes but we identified 21 so far unrecognized genes truly responsive only to the living plant. Among them we found several uncharacterized secondary metabolite gene clusters and putative membrane proteins with signaling functions. Interestingly, most of the downregulated transcripts potentially code for proteins with unknown functions making this category highly attractive for future research. It is not unreasonable to expect that some of these novel active-plant induced or repressed genes code for novel pathogenicity factors promoting the infection process or targeting of the plant defense system. Further studies by inactivation and overexpression of these genes will probably shed new light on the *F. graminearum*-wheat interaction process during the pathogenic process and it will also be interesting to see which of those genes are altered in their expression profiles in the epigenetic mutants we are studying in parallel.

## Materials and methods

### *F. graminearum* strain maintenance, axenic culturing, and spore production

The *F. graminearum* Ph-1 wildtype (FGSC 9075, NRRL 31084) applied in this study was maintained on *Fusarium* minimal medium (FMM) agar plates according to standard methods (Reyes-Dominguez et al., 2012). Details on cultivation conditions and media are given in Datasheet 8 in Supplemental Materials and Methods.

### Wheat infection experiment

The highly susceptible cultivar Remus (pedigree: Sappo/Mex//Famos; Buerstmayr et al., 2002) was used in this study. On average 20 spikelets per wheat head were inoculated by pipetting 20,000 macroconidia (10 μL of a 2 ^*^ 10^6^ spores/ mL suspension) on the reproductive part between the lemma and palea of the two basal florets during anthesis (resulting in 8 ^*^ 10^5^ spores per wheat head). Mock ears were inoculated the same way, but using water instead of spore suspension. Three ears were inoculated on the living plant representing pathogenic growth of the fungus and three ears, which were cut off the plant and shock-frozen in liquid N prior to spore application, were inoculated as “non-response” control from the wheat side representing saprophytic growth of the fungus. After inoculation the wheat heads on the living plants were covered with moistened plastic bags for the first 24 h to provide high humidity. The inoculated dead wheat heads were placed in glass petri dishes (Ø 140 mm; h 20 mm). Incubation conditions were set at 20⋅C, 50% relative humidity during daytime and 18⋅C, 50% humidity during night with a 16 h photoperiod. Harvesting was performed 3 and 5 dai by freezing the plant material in liquid nitrogen. Only palea and lemma of the inoculated florets were sampled including the respective part of the rachis. Additionally completely untreated wheat heads were sampled. After freezing in liquid nitrogen samples were stored at −80⋅C.

Cell disruption of the collected plant material was performed in the Retsch® Mixer Mill MM 301. The 50 mL steel grinding jars were used together with the 25 mm Ø steel balls (one ball per grinding jar). Before sample loading the grinding jars/ steel balls were baked at 270⋅C for 1 h, left at room temperature for 1 h and then pre-cooled overnight at −80⋅C. Milling was done at 30 Hz and room temperature for 20 s.

Independent RNA-extractions of three distinct pathogenic and three distinct saprophytic inoculated wheat heads harvested 3 dai were delivered for RNA-Seq analysis to the VetCORE—Facility for Research [University of Veterinary Medicine Vienna (VUW), Veterinärplatz 1, A-1210 Vienna, Austria].

### Chemical analysis of ornithine and sugar contents in culture supernatants

The levels of ornithine, fructose, glucose and sucrose in *Fusarium* culture supernatants were determined by applying a recently published GC-MS method (Warth et al., 2015). A concise description of the applied method and modifications is given in Datasheet 8 in Supplementary Material.

### Chemical analysis of nitrate content in culture supernatants

In the assay 100 μL culture filtrate or standard solution were mixed with 100 μL of acidic VCl_3_ (50.9 mM Vanadium(III) chloride [Sigma 2087272], 1 M HCl), 50 μL Griess Reagent I (N-(1-Naphthyl)ethylenediamine dihydrochloride [Sigma 33461]), and 50 μL Griess Reagent II (58 mM Sulfanilamide *alias* Amino-4 benzénesulfonamide [VWR 21156-237], 3 M HCl) incubated at 37⋅C for 1 h followed by measurement of extinction at 540 nm. Culture filtrates were diluted 1: 1000 prior to the assay and as standard solutions liquid FMM media (Reyes-Dominguez et al., 2012) was prepared containing 0.2125% (w/v) NaNO_3_ (25 mM) and diluted to final nitrate concentrations of 50, 40, 30, 20, and 10 μM.

### Chemical analysis of secondary metabolites culture supernatants

In case of the axenic minimal media cultures 1 mL of each culture filtrate was directly analyzed by liquid chromatography/electrospray ionization-tandem mass spectrometry (HPLC/ESI-MS/MS) as previously described (Sulyok et al., 2006; Vishwanath et al., 2009). A more detailed description of the applied method and modifications is given in Datasheet 8 in Supplementary Material.

### Chromosomal DNA and RNA extraction and RT-qPCR quantification of infection rate

#### DNA and RNA extraction and measurement of infection rates

Details for the standard procedures of DNA and RNA extractions are given in Datasheet 8 in Supplemental Materials and Methods. The measurement of infection rates is based on a qPCR method that quantifies the proportion of fungal chromosomal DNA (chrDNA) within the fungus/wheat DNA mixture (Brunner et al., 2009). The qPCR protocol was set up as it was described by Brunner et al. (2009) with following modifications: The external standard curve for the *TRI5* assay was generated by analyzing four-fold dilution series of *F. graminearum* DNA from pure fungal cultures starting with a concentration of 110 ng/μL (110, 27.5, 6.88, 1.72, 0.43 ng/μL), diluted in sterile water. The external standard curve for the *EF-G* assay was still done by measuring two-fold dilution series of wheat DNA starting from 100 ng/μL, but going down till 6.25 ng/μL (100, 50, 25, 12.5, 6.25 ng/μL), diluted in sterile water. For a single TaqMan reaction 2 μL template DNA was mixed with 7.5 μL Kapa Probe Fast qPCR Universal 2x Mastermix (Peqlab 07-KK4701), 4.78 μL sterile water, 0.24 μL dual-labeled probe, forward and reverse primer (each 100 pmol/ μL). Used primers and probes can be found in Brunner et al. (2009). The RT-qPCR analysis was performed on a BIORAD IQ™5 Multicolor Real-Time PCR Detection System. Cycling conditions included a single initial step at 95⋅C for 1 min 50 s, followed by 45 cycles of 95⋅C for 15 s, 52⋅C for 20 s, and 60⋅C for 15 s for the *TRI5* assay. In case of the *EF-G* assay the initial denaturation was set at 95⋅C for 1 min 50 s, followed by 45 cycles of 95⋅C for 15 s, 57⋅C for 20 s, and 62.5⋅C for 20 s.

### RNA extraction and RT-qPCR analysis

RNA isolation was performed using RNeasy Plant Mini Kit (Qiagen, 74904) following the instructions of the manufacturer with one modification and one extension: Instead of eluting RNA once in 30 μL RNase-free water, elution was performed in 2 × 20 μL RNase-free water with a 3 min standing period at room temperature after each water application to the column. Additionally on-column DNase digestion was done during the extraction protocol by using RNase-Free DNase Set (Qiagen, 79254). RNA quantity was first checked on the NanoDrop 2000c Spectrophotometer (Thermo Scientific). Afterwards RNA quantity and integrity was validated by using the Agilent RNA 6000 Nano Kit on an Agilent 2100 Bioanalyzer machine following the instructions of the provider. The cDNA Synthesis was carried out with the RevertAid H Minus First Strand cDNA Synthesis Kit (Thermo Scientific, # K1632) using random hexamer primer and following the manufacturer's protocol. Details on procedure and conditions for cDNA synthesis and determination of infection rates are given in Datasheet 8 in Supplemental Materials and Methods.

### RNA-seq mapping and quantification

The genome of *F. graminearum* and FGDB annotation version 3.2 was retrieved from http://www.helmholtz-muenchen.de/en/ibis/institute/groups/fungal-microbial-genomics/resources/index.html (Wong et al., 2011). RNA-seq reads were mapped on the reference genome using tophat2 (v2.0.8). The interval for allowed intron lengths were set to min 20 nt and max 1 kb (Trapnell et al., 2009; Kim et al., 2013). We used cufflinks to determine the abundance of transcripts in FPKM (Fragments Per Kilobase of exon per Million fragments mapped) and integrated the biological replicates using cuffdiff (Trapnell et al., 2010, 2012). The gene models were included as raw junctions. Genes with a minimum of two-fold increase or decrease in expression (|log2 of the FPKM values +1| ≥ 1) between the two experimental conditions were considered as regulated. Significant differential regulated genes of no functional annotation were manually re-visited. All gene models which were based on all current evidences including RNA-seq data (this study and unpublished) considered as spurious ORFs, were omitted from the downstream analysis. All data are available at NCBI GEO under the accession number GSE72124.

### Functional classification

Genes with a two-fold increase or decrease in expression between the two experimental conditions were analyzed for overrepresented functions. We therefore used the FunCat catalog of protein function (Ruepp et al., 2004) in combination with Fisher's exact test (Fisher, 1922) and the MGSA-R package (Bauer et al., 2010). Resulting *p*-values were corrected for multiple testing using the Benjamini Hochberg procedure (Benjamini and Hochberg, 1995).

### Prediction of putative secreted proteins

We computed secreted proteins in a pipeline approach. First we filtered on proteins targeted as secreted by TargetP (Emanuelsson et al., 2007) with an RC-score less than four. To add non-classically secreted proteins we selected on predicted secreted proteins using SecretomeP (Bendtsen et al., 2004) with a cutoff score of 0.65. This set of proteins was further filtered for those which are predicted as extracellular by Wolfpsort (Tamura and Akutsu, 2007). To exclude extracellular, membrane bound proteins we utilized TMHMM (Krogh et al., 2001) for transmembrane domain prediction and excluded proteins with more than one predicted transmembrane domain.

### Extracting factors from combinations

Factor dependent transcription was calculated as follows: Factor AP was calculated according to formula AP = [patho] − [(sapro + axo)/2], where patho, sapro, and axo represent log2(FPKM) values of the experiments pathogenic- saprophytic- and axenic- growth. Similarly, PP was calculated as PP = [(sapro + patho)/2] − [axo] and DI = [(axo + patho)/2] − [sapro]. Means and standard deviation were calculated by first order Taylor expansion in R. To determine if a gene is affected by a single factor *t*-tests between the groups of log2(FPKM) values between the specific contrasts, also indicated by square brackets, were calculated like for AP: patho vs. sapro & axo; PP: sapro & patho vs. axo; and DI: axo & patho vs. sapro.

The reasoning behind formula AP = [patho] − [(sapro + axo)/2]: patho is the only condition where a living plant is present so any difference between patho and the combination of sapro and axo is likely down to active plant action. PP = [(sapro + patho)/2] − [axo]: in both experiments sapro and patho is plant material but not in axo, so a deviation from zero may be cause by the plant. DI = [(axo + patho)/2] − [sapro]: both axo and patho experimental setups provide limited supply with nutrients vs. in sapro setup strong growth was observed what hints toward satisfactory supply. To determine if a factor is singly/mainly responsible we performed *t*-tests as described above.

## Author contributions

SB, IM, ML, and JS planned and performed fungal and plant experiments. HB, SB, CS, MM, UG, and JS analyzed and interpreted transcriptome data. BW, MS, and RS analyzed and interpreted metabolomic data. SB, HB, and JS wrote the main parts of the manuscript with contribution from all other authors.

### Conflict of interest statement

The authors declare that the research was conducted in the absence of any commercial or financial relationships that could be construed as a potential conflict of interest.
