# Supplementary material for: Comparison of Fusarium graminearum Transcriptomes on Living or Dead Wheat Differentiates Substrate-Responsive and Defense-Responsive Genes
Source: Front Microbiol. 2016 Jul 26;7:1113. doi: 10.3389/fmicb.2016.01113 (PMC4960244; doi:10.3389/fmicb.2016.01113)
Supplement: Supplementary file 1 [file DataSheet8.pdf]

Boedi et al.,

Supplemental Material and Methods

F. graminearum strain maintenance, axenic culturing and spore production

The *Fusarium graminearum* Ph-1 wildtype (FGSC 9075, NRRL 31084) was maintained on *Fusarium* minimal medium (FMM) agar plates according to standard methods (Reyes-Dominguez *et al.*, 2012). Details on cultivation conditions and media are as follows: Macroconidia production prior to inoculation of the axenic medium was done in mung bean medium (Reyes-Dominguez *et al.*, 2012). Approx. 1 cm^2 of *Fusarium* mycelium grown on a FMM agar plate was cut into small pieces and transferred to 75 mL mung bean medium within a 500 mL baffled flask. After incubation at 20 °C and 95 rpm for three days the resulting spore suspension was filtrated through glass wool to remove fungal mycelia. After precipitation of macroconida at 1811 *g and 4 °C for 5 minutes, the mung bean culture supernatant was replaced by autoclaved ddH_2O. Axenic Media culturing was performed in liquid FMM (Reyes-Dominguez *et al.*, 2012) and in liquid FMM containing instead of 0.2% (w/v) $NaNO_3$ (23.5 mM) 0.843% (w/v) L-Ornithine monohydrochloride (5mM; Sigma O2375). Before autoclaving pH 6.5 was adjusted using NaOH. 50 mL axenic medium was inoculated with 0.5 million macroconidia and incubated at 20 °C in the dark without shaking. The inoculated media were incubated in square petri dishes (120x 120x 17 mm; Greiner bio-one 688102) which were placed in a plastic box on racks with a layer of water underneath to facilitate humidity. Filtration of cultures was performed through Miracloth tissue (Millipore 475855-1R). Of the axenic media samples two independent biological repetitions each comprising two technical repetitions were analyzed for secondary metabolite production and transcript levels by RT-qPCR analysis; and one sample of each biological repetition of the cultivations on L-Ornithine was delivered for RNA-Seq analysis to the VetCORE – Facility for Research (University of Veterinary Medicine Vienna (VUW), Veterinärplatz 1, A-1210 Vienna, Austria). In case of the wheat infection experiment spores were obtained in liquid SNA medium (0.1 % (w/v) KH_2PO_4, 0.1 % (w/v) KNO_3, 0.05 % (w/v) $MgSO_4.7H_2O$, 0.05 % (w/v) KCl, 0.02 % (w/v) Glucose, 0.02 % (w/v) Saccharose, 1 % (w/v) Hefeextrakt, 0.06 % (v/v) 1N NaOH; autoclaved at 121 °C for 20 minutes). A FMM agar block comprising an area of approx. 1 cm^2 covered with *Fusarium* mycelia was cut into (approx. 5) small pieces and transferred to 70 mL liquid SNA medium within a 500 mL baffled flask. The sporulation cultures were incubated at room temperature and 120 rpm for 6 days and afterwards filtrated through milk filters (Calgonit 200 Kleencare Hygiene GmbH, Ladenburg) to remove fungal mycelia. Spore precipitation was done at 212 *g and room temperature for 10 minutes before the macroconidia concentration was adjusted to 2* 10^6 spores/ mL by dilution with ddH_2O.

Chemical analysis of ornithine and sugar contents in culture supernatants

The levels of ornithine, fructose, glucose and sucrose in *Fusarium* culture supernatants were determined by applying a recently published GC-MS method (Warth *et al.*, 2015). In brief, 200 µL of culture supernatants were transferred into micro-inserts in GC/HPLC vials and evaporated overnight using a centrifugal evaporator (Labconco, Kansas City, MO) and subsequently put on the GC auto sampler (PAL LHX-xt, CTC Analytics, Carrboro, NC). An optimized online two step derivatization procedure was applied as follows: The samples were re-suspended in 40 µL methoxyamine hydrochloride (20 mg/mL pyridine, Sigma Aldrich, Vienna, Austria) and agitated for 90 min at 60°C. Thereafter, 50 µL N-methyl-N-trimethylsilyl trifluoroacetamide (MSTFA, Wagner Munz, Vienna, Austria) and 10 µL of the internal standard nonadecanoic acid methylester (370 mg/L, Sigma Aldrich, Vienna, Austria) were added before the vial was again agitated at 60°C for 60 min. For chromatographic separation and detection of analytes an Agilent 7890A gas chromatograph coupled to a 5975C inert XL MSD detector (Agilent, Waldbronn, Germany) was applied. The liquid sample (1 µL) was injected into the split/splitless injector at 250°C in split mode and using a split of 25:1. An HP5-ms column (30 m x 0.25 mm x 0.25 µm; Agilent Technologies, Waldbronn, Germany) was operated at a constant flow of 1 mL/min helium. After keeping the oven at 50°C for 2 min, it was heated to 310°C (10°C/min) and kept at this temperature for 15 min while the MSD interface was kept at 290°C throughout the run. The EI source was kept at 230°C and the MSD quadrupole at 150°C. Raw GC-MS data files were acquired by ChemStation and evaluated using the MassHunter software (both from Agilent Technologies).

Chemical analysis of secondary metabolites culture supernatants

In case of the axenic minimal media cultures 1 mL of each culture filtrate was directly analyzed by liquid chromatography/ electrospray ionization-tandem mass spectrometry (HPLC/ESI-MS/MS) as previously described (Sulyok *et al.*, 2006, Vishwanath *et al.*, 2009). (Sulyok *et al.*, 2006)In case of the wheat infection experiment 1 mL extraction solvent (MeOH : H_2O 3:1 (v/v); +0.1 % HCOOH) was added to approximately 100 mg of milled cell material (wet weight). After solution addition samples were vortexed for 10 seconds and treated in an ultrasonication bath (Branson 3210) for 15 minutes at room temperature. After centrifugation at 41 627 *g and 4 °C for 10 minutes 700 µL aliquots were transferred into 1.5 mL HPLC vials. After addition of 350 µL dilution solvent (H_2O + 0.1 % HCOOH) the HPLC vials were closed, vortexed for 10 seconds and the samples were analysed by HPLC/ESI-MS/MS as

previously described (Vishwanath *et al.*, 2009, Sulyok *et al.*, 2006). Quantified metabolite levels were normalized due to the input sample weights.

DNA and RNA extraction

For chromosomal DNA extraction 900 µL – 700 µL CTAB-Buffer (100 mM Tris-HCl pH 7.5; 700 mM NaCl; 50 mM EDTA pH 8.0; 140 mM β-Mercaptoethanol; 1 % (w/v) Cetyltrimethylammonium-bromid (CTAB; Carl Roth 9161.1) was added to ~ 25 mg of milled cell material. CTAB-Buffer was always fresh made and heat up to 60 °C – 65 °C before addition of β-Mercaptoethanol and CTAB. Samples were incubated at 65 °C and moderate shaking for 60 minutes. Afterwards samples were cooled down to room temperature and 450 µL – 350 µL CI (chloroform: isoamyl alcohol 24:1 (v/v)) were added. The two phases were mixed by gentle inversion for 5 minutes prior to phase separation by centrifugation at 3500 *g, room temperature. 800 µL – 650 µL of the upper phase were transferred to a new tube and RNA was digested by addition of 60 µg RNase A (Invitrogen™ 12091-039) and incubation at room temperature for 30 minutes. Afterwards precipitation was done by addition of one volume Isopropanol, gently inverting the tubes and centrifugation at 15 871 *g, room temperature. The supernatant was poured off and the pellet was washed first by addition of 200 µL wash 1 solution (76 % (v/v) ethanol, 2.5 M NaOAc). The tubes were gently inverted at room temperature for 5 minutes before the pellet was spinned down by centrifugation at 15 871 *g for 5 minutes at room temperature. The washing procedure was repeated by using wash 2 solution (76 % (v/v) ethanol, 10 mM NaOAc) and after centrifugation the supernatant was carefully removed by using the pipette. The pellet was dried for 10 minutes at 60 °C before it was resuspended in 100 µL of 0.05x TE-Buffer (0.5 mM Tris-HCl pH 7.5; 0.05 mM EDTA pH 8.0) and left in moderate shaking at room temperature to enhance pellet dissolving. The total DNA concentration was determined using the NanoDrop 2000c Spectrophotometer (Thermo Scientific) and the samples higher concentrated as 97 ng/ µL were diluted to a final concentration of 50 ng/µL in sterile water (DNA isolations lower than 97 ng/ µL were applied undiluted to the qPCR reactions).

cDNA synthesis and RT-qPCR quantification of infection rate

The cDNA synthesis started with 1 µg input RNA; after the procedure a 1:10 dilution of the reverse transcription reaction product was done to gain enough template volume for the following qPCR reactions. The qPCR analysis was performed on a BIORAD MyiQ™ Single Color Real-Time PCR Detection System. For a single qPCR reaction 5 µL template cDNA

was mixed with 10 µL iQ™ SYBR® Green Supermix (Biorad, 170-8887), 4.2 µL sterile water and 0.4 µL forward and reverse primer (each 10 pmol/ µL). The cycling conditions were set as follows: 50 °C for 2 minutes, 95 °C for 2 minutes followed by 40 cycles of 95 °C for 15 seconds, the corresponding annealing temperature for 30 seconds and 72 °C for 30 seconds. Afterwards melting curve analysis was done starting at 95 °C for 30 seconds, going down to 55 °C for 30 seconds and then recording the melting profile of the PCR products by increasing the temperature from 55 °C by 0.5 °C in 80 15 seconds intervals.

To estimate to which extent the ratio of fungal chrDNA to wheat chrDNA correlates to the RNA proportion qPCR analysis of GAPDH transcripts was done. Primers Fus_GAPDH_qPCR_fwd (5´-CGTCAACGGCAAGACCATCAAGTT-3´) and Fus_GAPDH_qPCR_rev (5´-CCCTTCTCGAGGCGAACAGTCAAG-3´) were chosen from Skov *et al.* (2004) to quantify the *F. graminearum* glyceraldehyde-3-phosphate dehydrogenase transcript (FGSG_16627). Primers Wheat_GAPDH_qPCR_fwd (5´-TCACCACCGACTACATGACC-3´) and Wheat_GAPDH_qPCR_rev (5´-ACAGCAACCTCCTTCTCACC-3´) were chosen from Charkazi *et al.* (2010) to quantify the *Triticum aestivum* glyceraldehyde-3-phosphate dehydrogenase gene (accession no. EF592180) transcript. For each primer pair a separate external standard curve was generated by qPCR analysis of a defined PCR product that was amplified with external primers once from cDNA of an axenic *Fusarium graminearum* culture and once from cDNA of a mock wheat ear. Via the molecular mass of the respective PCR product copy numbers of the molecule from ranging from 10^6 to 10^2 in tenfold dilution steps were reached in the standard dilutions. The PCR product that was used as standard for the *Fusarium* GAPDH qPCR analysis was amplified by primers Fus_GAPDH_STD_fwd (5´-AAGTACGACTCTTCCCACGGTA-3´) and Fus_GAPDH_STD_rev (5´-CTCAGAGGCCTCCTTGATGA-3´). The PCR product that was used as standard for the wheat GAPDH qPCR analysis was amplified by primers Wheat_GAPDH_STD_fwd (5´-GATCGGTATCAACGGTTTCG-3´) and Wheat_GAPDH_STD_rev (5´-TAGTGGTGCAGCTAGCGTTG-3´).

In addition to RNA-Seq analysis RT-qPCR measurement of following genes were done using *F. graminearum* chrDNA from pure fungal cultures as standard, ranging from a concentration of 11 ng/ µL to 1.1 $*10^{-3}$ ng/ µL in tenfold dilution steps in sterile water: From the core *TRI5*-cluster (Kimura *et al.*, 2003, Desjardins *et al.*, 1993, Hohn *et al.*, 1993) the trichodiene synthase (sesquiterpene cyclase) gene *TRI5* (FGSG_03537) (Hohn & Beremand, 1989) was analyzed with primers Tri5_ORF_fwd (5´-GGAGAAGCTCACCCAGGAAACC-3´) and Tri5_ORF_rev (5´-AATCTCATGGAGGCGGTATCGAG-3´); and the zinc finger transcription factor encoding *TRI6* gene (FGSG_16251) (Proctor *et al.*, 1995, Hohn *et al.*, 1999) was

analysed with primers Tri6_ORF_fwd (5´-GACTTCGCGAACCCGGCTAT-3´) and Tri6_ORF_rev (5´-CGTCCGCTTTCAAAGACTGTGG-3´). From the biosynthetic pathway of butenolide the putative benzoate 4-monooxygenase cytochrome P450 gene *FGSG_08079* and the putative regulatory gene *FGSG_08080* whose product contains a Zn(2)–C6 fungal-type DNA-binding domain (Harris *et al.*, 2007) were analysed with primers FGSG_08079_ORF_fwd (5´-CAGGCCGAATTGGATGAAGC-3´) and FGSG_08079_ORF_rev (5´-AGACCGAGGCTGATGGTGGA-3´) as well as FGSG_08080_ORF_fwd (5´-CATTGACTGGGGCGACATGA-3´) and FGSG_08080_ORF_rev (5´-GTTGGTGCGGCAAAATCTCC-3´), respectively. Regarding culmorin biosynthesis we analysed the longiborneol synthase gene *CLM1* (FGSG_10397) (McCormick *et al.*, 2010) using primers CLM1_ORF_fwd (5´-CCCCCTTGTTGCGAATCTGA-3´) and CLM1_ORF_rev (5´-TCCCGTTCCCGGATATACCA-3´). As additional reference gene β-tubulin (FGSG_09530) transcript levels were quantified using primers FG_tubB_ORF_fwd (5´-GTCTCCGTTTCCCCGGTCAG-3´) and FG_tubB_ORF_rev (5´-GCTGGGTGAGCTCAGGAACG-3´).

References Supplemental Material

Charkazi, F., S.S. Ramezanpour & H. Soltanloo, (2010) Expression Pattern of Two Sugar Transporter Genes (SuT4 and SuT5) under Salt Stress in Wheat *Plant Omics* **3**: 194-198.

Desjardins, A.E., T.M. Hohn & S.P. McCormick, (1993) Trichothecene biosynthesis in *Fusarium* species: chemistry, genetics, and significance. *Microbiological reviews* **57**: 595-604.

Harris, L.J., N.J. Alexander, A. Saparno, B. Blackwell, S.P. McCormick, A.E. Desjardins, L.S. Robert, N. Tinker, J. Hattori, C. Piche, J.P. Schernthaner, R. Watson & T. Ouellet, (2007) A novel gene cluster in *Fusarium graminearum* contains a gene that contributes to butenolide synthesis. *Fungal genetics and biology : FG & B* **44**: 293-306.

Hohn, T.M. & P.D. Beremand, (1989) Isolation and nucleotide sequence of a sesquiterpene cyclase gene from the trichothecene-producing fungus *Fusarium sporotrichioides*. *Gene* **79**: 131-138.

Hohn, T.M., R. Krishna & R.H. Proctor, (1999) Characterization of a transcriptional activator controlling trichothecene toxin biosynthesis. *Fungal genetics and biology : FG & B* **26**: 224-235.

Hohn, T.M., S.P. McCormick & A.E. Desjardins, (1993) Evidence for a gene cluster involving trichothecene-pathway biosynthetic genes in *Fusarium sporotrichioides*. *Current genetics* **24**: 291-295.

Kimura, M., T. Tokai, K. O'Donnell, T.J. Ward, M. Fujimura, H. Hamamoto, T. Shibata & I. Yamaguchi, (2003) The trichothecene biosynthesis gene cluster of *Fusarium graminearum* F15 contains a limited number of essential pathway genes and expressed non-essential genes. *FEBS letters* **539**: 105-110.

McCormick, S.P., N.J. Alexander & L.J. Harris, (2010) *CLM1* of *Fusarium graminearum* encodes a longiborneol synthase required for culmorin production. *Applied and environmental microbiology* **76**: 136-141.

Proctor, R.H., T.M. Hohn, S.P. McCormick & A.E. Desjardins, (1995) *Tri6* encodes an unusual zinc finger protein involved in regulation of trichothecene biosynthesis in *Fusarium sporotrichioides*. *Applied and environmental microbiology* **61**: 1923-1930.

Reyes-Dominguez, Y., S. Boedi, M. Sulyok, G. Wiesenberger, N. Stoppacher, R. Krska & J. Strauss, (2012) Heterochromatin influences the secondary metabolite profile in the plant pathogen *Fusarium graminearum*. *Fungal genetics and biology : FG & B* **49**: 39-47.
Skov, J., M. Lemmens & H. Giese, (2004) Role of a *Fusarium culmorum* ABC transporter (FcABC1) during infection of wheat and barley. *Physiological and Molecular Plant Pathology* **64**: 245-254.
Sulyok, M., F. Berthiller, R. Krska & R. Schuhmacher, (2006) Development and validation of a liquid chromatography/tandem mass spectrometric method for the determination of 39 mycotoxins in wheat and maize. *Rapid communications in mass spectrometry : RCM* **20**: 2649-2659.
Vishwanath, V., M. Sulyok, R. Labuda, W. Bicker & R. Krska, (2009) Simultaneous determination of 186 fungal and bacterial metabolites in indoor matrices by liquid chromatography/tandem mass spectrometry. *Analytical and bioanalytical chemistry* **395**: 1355-1372.
Warth, B., A. Parich, C. Bueschl, D. Schoefbeck, N.K. Neumann, B. Kluger, K. Schuster, R. Krska, G. Adam, M. Lemmens & R. Schuhmacher, (2015) GC-MS based targeted metabolic profiling identifies changes in the wheat metabolome following deoxynivalenol treatment. *Metabolomics* **11**: 722-738.
