# Supplementary material for: Comparison of Fusarium graminearum Transcriptomes on Living or Dead Wheat Differentiates Substrate-Responsive and Defense-Responsive Genes
Source: Front Microbiol. 2016 Jul 26;7:1113. doi: 10.3389/fmicb.2016.01113 (PMC4960244; doi:10.3389/fmicb.2016.01113)
Supplement: Supplementary file 2 [file Image1.pdf]

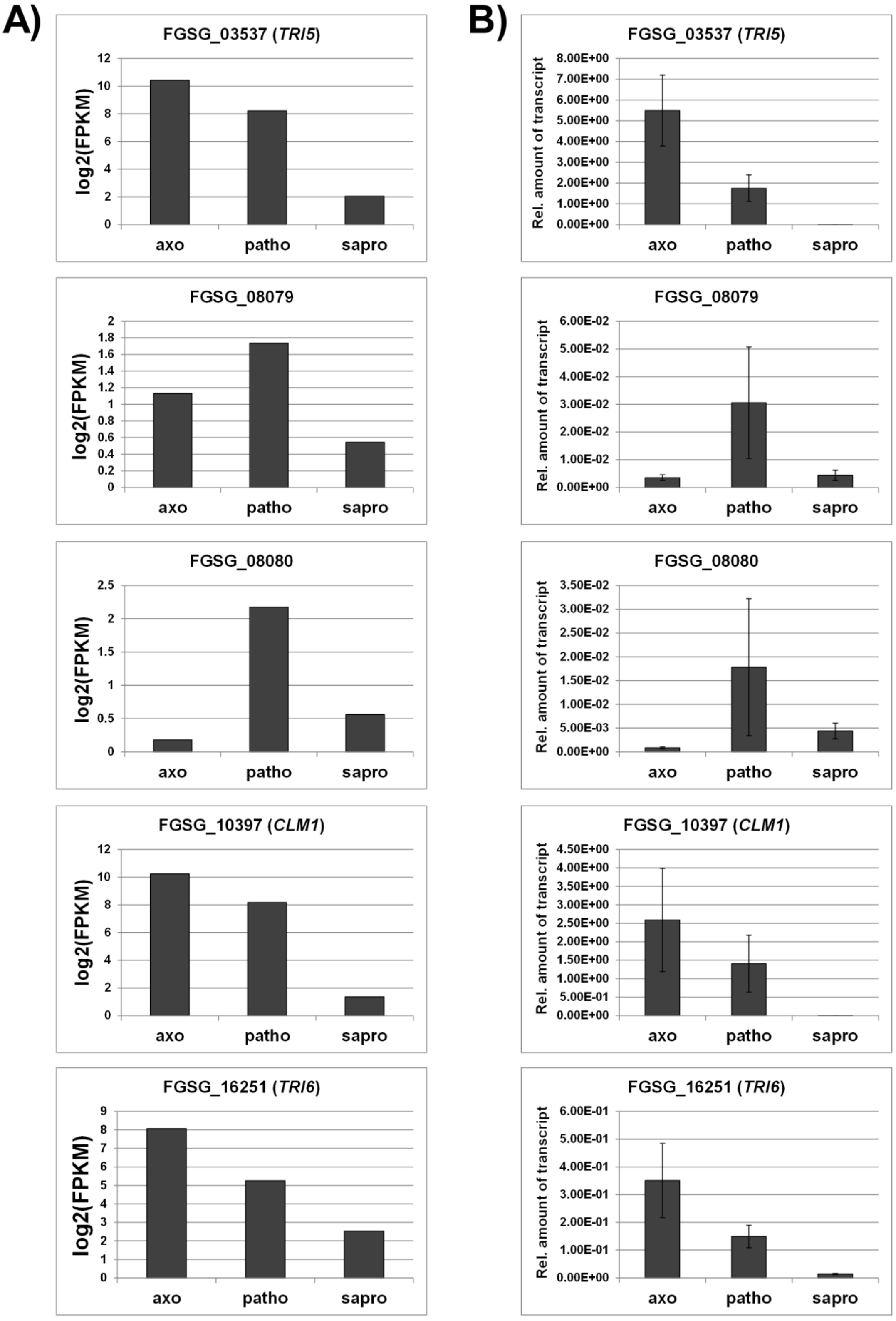

Figure S1: Verification of relative transcript abundances found in RNA-seq analysis (**A**) by RT-qPCR (**B**). Details of RNA-seq analysis and primers as well as conditions for RT-qPCR are described in Materials and Methods (Patho... pathogenic growth; sapro... saprophytic growth; axo... axenic growth on L-ornithine).
