# Supplementary material for: Comparison of Fusarium graminearum Transcriptomes on Living or Dead Wheat Differentiates Substrate-Responsive and Defense-Responsive Genes
Source: Front Microbiol. 2016 Jul 26;7:1113. doi: 10.3389/fmicb.2016.01113 (PMC4960244; doi:10.3389/fmicb.2016.01113)
Supplement: Supplementary file 3 [file Image2.pdf]

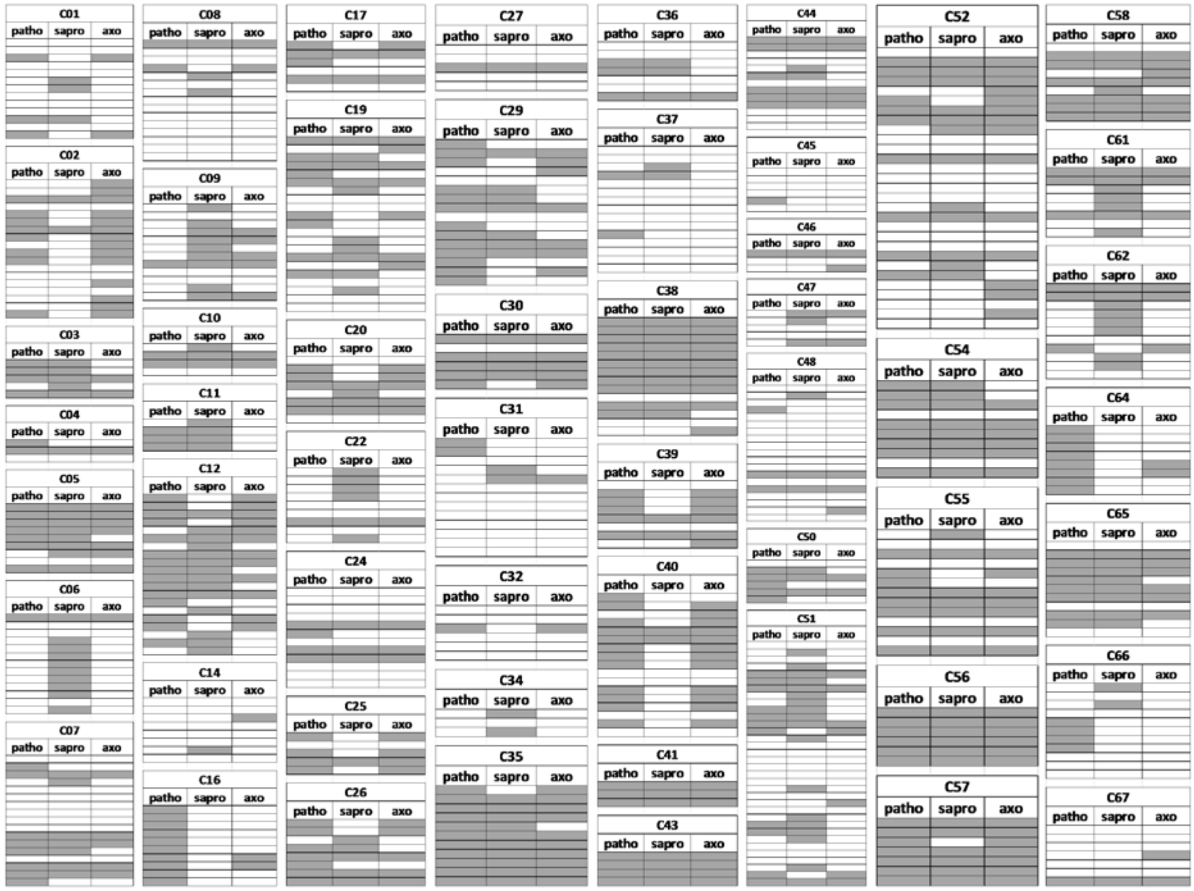


**Figure S2:** Schematic overview illustrating expression distribution of genes within putative biosynthetic clusters of unknown metabolites (Sieber et al. 2014) above the log2(FPKM) thresholds demarcated by the 65 % of highest expressed genes within the respective growth condition, which are 1.99 in case of pathogenic (patho), 2.46 in case of saprophytic (sapro) and 1.92 in case of axenic (axo) growth. Three horizontal fields in one line represent one gene and grey indicates expression above the 65 % threshold.
