# Supplementary material for: Comparison of Fusarium graminearum Transcriptomes on Living or Dead Wheat Differentiates Substrate-Responsive and Defense-Responsive Genes
Source: Front Microbiol. 2016 Jul 26;7:1113. doi: 10.3389/fmicb.2016.01113 (PMC4960244; doi:10.3389/fmicb.2016.01113)
Supplement: Supplementary file 4 [file Table1.pdf]

Table S1: Quantification of L-ornithine, nitrate and sugar compounds in minimal media supernatants not inoculated (n. i.) and 3 days after inoculation (3 dai) with *F. graminearum* Ph-1 wild type spores. Values in brackets represent standard deviations of two independent biological repetitions. Calculated sucrose proportions are based on measured sugar contents. The limit of detection (LOD) within the ornithine measurement is given by 0.04 mM.

	**L-ornithine [mM]**	**nitrate [mM]**	**fructose [mM]**	**glucose [mM]**	**sucrose [mM]**	**sucrose left [%]**	**sucrose hydrolysed [%]**	**sucrose consumed [%]**
**n. i.**	7.1 (±0.4)	22 (±2)	2.0 (±0.5)	2.4 (±1.0)	118.6 (±1.8)	100		
**3 dai**	<LOD	15 (±2)	31.4 (±2.9)	31.6 (±2.0)	51.3 (±1.7)	43	27	30
